# *Anshen Bunao* syrup as a potential anti-aging agent: mechanistic insights and pharmacological evidence

**DOI:** 10.3389/fragi.2026.1768671

**Published:** 2026-03-10

**Authors:** Yuanfang Sun, Qi Xia, Ruolan Wu, Gang Liu, Yongkuan Wang, Shikai Yan, Huizi Jin, Xiuyun Zhang, Xue Xiao, Shasha Li

**Affiliations:** 1 Institute of Traditional Chinese Medicine, Guangdong Pharmaceutical University, Guangzhou, China; 2 The Second Clinical College of Guangzhou University of Chinese Medicine, Guangzhou, China; 3 Shanghai Key Laboratory for Molecular Engineering of Chiral Drugs, School of Pharmacy, Shanghai Jiao Tong University, Shanghai, China; 4 Jilin Aodong Yanbian Pharmaceutical Co., Ltd., Yanbian, China

**Keywords:** aging, Anshen Bunao syrup, inflammation, metabolomics, oxidative stress, transcriptomics

## Abstract

**Background:**

*Anshen Bunao* Syrup (ABS) is a commonly used traditional Chinese patent medicine for insomnia and amnesia, and also has the potential to anti-aging but lacks scientific support of pharmacological and mechanism research. This study aims to investigate the anti-aging effect of ABS and its underlying mechanism.

**Methods:**

Chemical constituents of ABS were analyzed by LC/MS. ABS effects were assessed on d-galactose induced aging rat model through open field tests, histopathological examinations, senescence-associated secretory phenotypes and biochemical assay. Integrated analysis of transcriptomics and metabolomics was conducted to identify differentially expressed genes and metabolites and to elucidate the potential mechanism.

**Results:**

159 chemical constituents in ABS were profiled and structurally presumed. ABS can significantly alleviate skin aging, neural damage and other aging related symptoms, and as well can reduce the expression of senescence-associated secretory phenotypes. Integrated transcriptomics and metabolomics revealed that ABS treatment modulates the expression levels of CYP2J10, CBR3, and LPAR3, and restores sphingolipid and arachidonic acid metabolism. ELISA results indicated that ABS reduces pro-inflammatory factor levels and relieve oxidative stress.

**Conclusion:**

ABS exerts anti-aging effects by regulating sphingolipid and arachidonic acid metabolism, thereby inhibiting inflammation and oxidative stress. This study is expected to provide research foundation for the development of ABS as a new anti-aging drug.

## 1 Introduction

Aging and aging related issues have become a major challenge for society, economy, and public health. World Health Organization projections indicate that by 2050, individuals aged 60 and older will outnumber those aged 10 to 24, with figures soaring to 2.1 billion vs. 2.0 billion (Rudnicka et al., 2020). Aging is the main risk factor for a series of chronic diseases such as Alzheimer’s disease, osteoporosis and cardiovascular disease, and is thus largely responsible for the ever-rising global incidence rate, mortality and healthcare costs.

The aging process is extremely complex. Till now, although people gradually realize that aging involves telomere attrition, mitochondrial dysfunction, loss of protein homeostasis, epigenetic changes, dysbiosis of the microbiota, chronic inflammation and so on (López-Otín et al., 2013; López-Otín et al., 2023), its mechanism is still unclear, which led to a huge challenge in the research and development of anti-aging drugs. At present, a series of compounds have been reported to have certain anti-aging effects, such as rapamycin (Blagosklonny, 2019), metformin (Zhang et al., 2023), senolytics (Lelarge et al., 2024), Spermidine (Madeo et al., 2018), and some of these drugs are already in clinical trials (Guarente et al., 2024). Anti-aging drugs require long-term use, and their safety assessment is particularly important; however, long-term safety data for current anti-aging drugs under development are generally lacking, and some drugs were proved to have safety issues. For example, rapamycin may cause dyslipidemia, elevated blood sugar, and increased resting heart rate (Johnson and Kaeberlein, 2016), and senolytics may prevent wound healing and tissue regeneration (Rad and Grillari, 2024), and long-term use of metformin may lead to lactic acidosis, and so on (Tarry-Adkins et al., 2021).

Developing anti-aging drugs based on western-drug research model seems to be in a dilemma. For decades, traditional Chinese medicine (TCM) has increasingly aroused research interest worldwide. The clinical efficacy and safety of TCM have definite advantages over western medicine, especially the principles of multi-component, multi-target and multi-mode which is particularly suitable for treating aging and other complex chronic diseases. At present, a large number of traditional Chinese medicinal materials were proved to have anti-aging effects (Wang et al., 2021), including *ginseng*, *astragalus*, *Epimedium*, *Salvia miltiorrhiza*, and some TCM formulas such as *Bazi Bushen* Capsules. There is great potential for the research and development of anti-aging drugs from TCM.

According to TCM theory, the essential characteristic of aging is kidney-esence deficiency, *Qi*-blood insufficient, heart-spirit malnutrition, and *zang-fu* organ functional decline. *Anshen Bunao* Syrup (ABS) is a commonly used traditional Chinese patent medicine for insomnia and amnesia, and is made from *Cervus nippon* Temminck, *Polygonum multiflorum* Thunb, *Epimedium brevicornu* Maxim, *Zingiber officinale* Rosc, *Glycyrrhiza uralensis* Fisch, *Ziziphus jujuba* Mill and Vitamin B1. ABS has effects of the kidney essence tonifying, *Qi* and blood nourishing, brain invigorating and spirit calming, which fully conforms to the core pathogenesis of aging. The major medicinal raw materials thereof have been reported to have anti-aging effects, including *Cervus nippon* Temminck (Li et al., 2021; Zhang et al., 2004; Wang, et al., 2019), *Polygonum multiflorum* Thunb (Liu et al., 2021), *Epimedium brevicornu* Maxim (Long et al., 2025); in addition, a cross-sectional study showed that intake of vitamin B1 was negatively associated with accelerated aging (Ma et al., 2024). *Glycyrrhizae Radix Zingiberis Rhizoma,* and *Jujubae Fructus* were also reported longevity-promoting properties in *Caenorhabditis elegans* (Ruan et al., 2016; Xu et al., 2022; Zhang et al., 2024; Li et al., 2021). Even though there is no official statement in the Pharmacopoeia, anti-aging is expected to be a new therapeutic indication for ABS, but lacks scientific support of pharmacological and mechanism research.

This study aims to investigate the anti-aging effect of ABS and its underlying mechanism. Chemical constituents of ABS were analyzed by LC/MS, and then behavioral testing, histological observations, and biochemical assays were employed to investigate the effects of ABS on aging in rat model. The mechanism was explored using an integrated analysis of transcriptomics and metabolomics. This study is expected to provide scientific support for the development of new indication of anti-aging for ABS.

## 2 Materials and methods

### 2.1 Chemicals and reagents

*Anshen Bunao* Syrup (lots: 2306220, 2306221, 2306222) was provided by Jilin Aodong Yanbian Pharmaceutical Co., Ltd*.* (Dunhua, Jilin, China). D-(+)-Galactose (lot: C14971412) and rapamycin were purchased from Macklin Biochemical Technology Co., Ltd. (Shanghai, China; lot: C14513337). The 0.9% sodium chloride injection (Guangdong Daxiang Pharmaceutical Co., Ltd, Guangzhou, Guangdong, China; lot: 230718501) was obtained from the pharmacy department of Guangdong Provincial Hospital of Chinese Medicine. Isofluorane was purchased from Shenzhen Ruiwode Life Technology Co., Ltd (Shenzhen, Guangdong, China). The 4% tissue cell fixative solution (Guangzhou Jingxin Biotechnology Co., Ltd, Guangzhou, Guangdong, China), anhydrous ethanol, xylene, neutral gum (Sinopharm Chemical Reagent Co., Ltd., Shanghai, China) and toluidine blue solution (Wuhan Pinofi Biotechnology Co., Ltd., Wuhan, Hubei, China) were used for Nissl staining. Chromatography-grade formic acid and chromatography-grade methanol were purchased from Thermo Fisher Scientific Inc. (MA, United States). Pure water for LC-MS analysis was purified using a Milli-Q water purification system (Millipore, MA, United States).

### 2.2 Qualitative analysis of chemical component

UPLC analysis was conducted on a Waters Acquity UPLC system equipped with solvent manager F12UPB023A, sample manager E12UPA754 and Acquity Console software (Waters, MA, United States). Chromatographic separation was performed on an Acquity UPLC HSS T3 analytical column (2.1 × 100 mm, 1.8 μm; Waters, MA, United States) at 30 °C. The mobile phase was composed of 0.1% (v/v) water-formic acid (A) and methanol (B) with the following gradient elution conditions: 0–3 min, 100% A; 3–11 min, 100%-72% A; 11–13 min, 72%-67% A; 13–18 min, 67%-50% A; 18–21 min, 50%-46% A; 21–32 min, 46%-27% A; 32–35 min, 27%-2% A; 35–37 min, 2% A. The flow rate was sustained at 0.3 mL/min with injection volume of 2 μL.

MS identification was carried out on a quadrupole time-of-flight mass spectrometer (TripleTOF® 5,600 System; AB SCIEX, MA, United States) with Analyst® TF 1.6 software (AB SCIEX, MA, United States). The parameters of ESI source were set as follows: ion source temperature, 500 °C; heated gas, 50 psi; nebulizer gas, 50 psi; curtain gas, 35 psi; collision energy (TOF MS), 10 V; collision energy (Product Ion) 40 ± 20 V. Full mass scan was set in the range of m/z 50–2000 Da. MS/MS data was acquired in information-dependent acquisition (IDA) mode.

### 2.3 Animals

All experiments were conducted in accordance with the protocol approved by the Ethics Committee of Guangdong Provincial Hospital of Chinese Medicine (No: 2023050). 60 Sprague-Dawley rats (8–10 weeks, male) were housed with *ad libitum* access to food and water under standard environmental conditions (8:00 AM-8 PM light cycle; 22 °C; 55% humidity). Rats were divided into 6 groups (n = 10) after acclimation for a week, including control group (CON), model group (MOD), positive control group (POS), ABS low-dose group (ASL), ABS medium-dose group (ASM) and ABS high-dose group (ASH). Rats in treatment groups were administered with ABS intragastrically (dose of 1.05, 2.10 and 4.20 mL/kg/d, respectively, for rats in ABL, ABM and ABH), and rats in POS group were administered with rapamycin (Selvarani et al., 2021; Stojic et al., 2023) intragastrically (0.5 mg/kg/d), while rats in CON and MOD were administered with water (1 mL/kg/d). After 2 weeks of administration, aging rat model began to be constructed. All rats were administered d-(+)-Galactose (120 mg/kg/d) subcutaneously except control rats, which were administered an equal volume of 0.9% sodium chloride injection.

### 2.4 Open field test

The open field test was conducted using a 5-min trial within a black opaque arena measuring 100 cm × 100 cm × 40 cm. Prior to the experiment, rats were acclimated to the testing environment for a period of 30–60 min. Relevant parameters, including the identification number, date, and status of each rat, were preconfigured in the Smart 3.0 software. Each rat was gently placed at the center of the arena from a consistent position and orientation to initiate the test, and its behavior was video-recorded to quantify total movement distance and mean speed using the software. To prevent olfactory interference, the arena was thoroughly cleaned with 75% ethanol and allowed to dry completely between each test.

### 2.5 Morris water maze test

The Morris water maze (MWM) test was often used to evaluate spatial learning and memory capacity (Yang et al., 2016). The maze consisted of a large circular pool (diameter 200 cm) full of 16 cm depth of water (26 °C) mixed with black paint. The pool was divided in four imaginary quadrants, and a circular platform (diameter 15 cm) was hidden 1 cm beneath the water surface at a fixed position. The experimenter always sat in the same place. During the training phase, rats underwent training over four consecutive days, with four trials per day and a 60-min interval between trials. Each rat was randomly introduced into the water from one of the four quadrants, facing the pool wall, and allowed to swim for 60 s to locate the hidden platform. Upon successfully finding the platform, the rat was permitted to rest on it for 10 s. If the rat failed to find the platform within 60 s, they were guided to the platform, allowing for a 10-s rest to ensure equal spatial information acquisition time. After the fourth learning day, rats were conducted out a probe trial to evaluate spatial retention memory. During the probe trial, the platform was removed, and the time spent in each quadrant, as well as the target latency were measured over a 60-s period with Smart v3.0 software.

### 2.6 Exclusion criteria

All 60 rats were subjected to open field test and Morris water maze test with 10 rats in each group. Exclusion criteria for the open field test were that the total movement distance of the rats was less than 20 m (rats barely moved throughout the entire trial) or more than 100 m (inaccurate software tracking caused by erratic jumping of rats, leading to falsely elevated distance values). Finally, the CON group included 9 rats, the MOD group included 10 rats, the ASL group included 9 rats, the ASM group included 7 rats, the ASH group included 6 rats, and the POS group included 8 rats.

Exclusion criteria in the Morris water maze included: a, rats excluded in the open field test; b, rats moved less than 10 m in the water maze test; c, rats jump out of the pool during the water maze test. Finally, the CON group included 8 rats, the MOD group included 8 rats, the ASL group included 9 rats, the ASM group included 5 rats, the ASH group included 6 rats, and the POS group included 8 rats.

### 2.7 Sample collection

All rats were anesthetized by continuous inhalation of isoflurane and their dorsal hair was carefully removed. Whole blood was taken from the abdominal aorta of rats, and the serum was separated by centrifugation at 3,500 rpm, 4 °C for 15 min, and stored at −80 °C. After rats were exsanguinated, both dorsal skin and brain were collected following 0.9% saline intracardial perfusion. Histological assessment was conducted in 3 rats/group, their dorsal skin and brain were immediately placed into 4% tissue cell fixative solution. The temporal cortex tissue was separated from brain, and immediately placed in liquid nitrogen.

### 2.8 Histopathological analysis

After 24 h, the samples are subjected to gradient dehydration for embedding in paraffin. The tissues are sliced into 4-μm thick sections. Nissl staining of brain slices was performed using the conventional method with a toluidine blue solution. According to routine protocols, the rats’ dorsal skin was stained with HE staining.

### 2.9 Biochemical indicators detection

Senescence-associated secretory phenotype (SASP) is a general term for a series of cytokines, such as proinflammatory cytokines, chemokines and proteases, which is a key feature of senescent cells. Therefore, SASP-related biochemical indicators were examined with Enzyme-linked immunosorbent assay (ELISA) kits, including interleukin-1β (IL-1β, ml037361), interleukin-6 (IL-6, ml064292), interleukin-10 (IL-10, ml037371), tumor necrosis factor-alpha (TNF-α, ml002859), transforming growth factor beta (TGF-β, ml107101), and matrix metallopeptidase 1 (MMP1, ml002968). As well, we measured C-reactive protein (CRP, ml002999) and oxidative stress-related indicators, likely malondialdehyde (MDA, ml093013), superoxide dismutase (SOD, ml092620) activity, and glutathione peroxidase (GSH-Px, ml107105) activity. The sample detected by ELISA is the cerebral cortex of rats, and kits were purchased from *Shanghai Enzyme-linked Biotechnology Co., Ltd*. (Shanghai, China). The assays were in strict accordance with the instructions of the ELISA kit.

### 2.10 Metabolomics

#### 2.10.1 UPLC-Q/TOF MS analysis

The serum of all the rats (n = 10 for each group) were used as test samples for metabolomics. Thawed serum (400 μL) was mixed with methanol (1,200 μL), then swirled for 2 min and centrifuged at 13,000 rpm for 15 min at 4 °C. The supernatant was filtered through a 0.22 μm microporous membrane and transferred to a new EP tube for UPLC-Q TOF/MS analysis.

Metabolomics was performed using an Acquity UPLC BEH C18 analytical column (2.1 mm × 100 mm, 1.7 μm). The eluent was water with 0.1% formic acid (solvent A) and methanol (solvent B). The solvent gradient was as follows: 0–15 min, 95%-80% A, 15–40 min, 80%-30% A, and 40–45 min, 30%-0 A. Elution was performed at a flow rate of 0.3 mL/min. MS is performed with an ESI interface in the positive mode, and the data acquisition modes were IDA continuum. The above MS system was used to carry out the plasma un-target metabolomics analysis, using the same parameters as the qualitative analysis.

#### 2.10.2 Data processing

The acquired raw data were processed on Progenesis QI version 2.4 (Waters Corp., MA, United States). Metabolite identification was performed using the primary and secondary MS information on acquired MS data and Human metabolite database (HMDB, http://www.hmdb.ca/). Supervised partial least squares discriminant analysis (PLS-DA) was performed using SIMCA-P+(version 13.0) software (Umetrics, Umeå, Sweden). Volcano plots were generated using MetaboAnalyst 6.0 (https://www.metaboanalyst.ca, updated: 2024.1.15). Potential biomarkers were screened by Volcano plots as a fold change value >1.5 and a P-value <0.05. GraphPad Prism software v 8.0 (GraphPad Software, CA, United States) was used to plot heat map.

#### 2.10.3 Pathway analysis

Pathway analysis was performed on potential biomarkers using MetaboAnalyst 6.0. The results were presented based on significance (hypergeometric test) and pathway impact (Relative-betweeness centrality) implemented in MetaboAnalyst 6.0.

### 2.11 Transcriptomics

#### 2.11.1 RNA extraction and library construction

Cortex samples of CON, MOD and ASH groups (n = 3 for each group) were delivered to Guangzhou Genedenovo Biotechnology Co., Ltd for RNA extracting, DNA library constructing and sequencing. Total RNA was extracted using a TRIzol total RNA extraction kit (Life technologies). RNA with a 260/280 absorbance ratio of 1.8–2.0 was considered high-quality RNA used for library construction and sequencing. The library was sequenced using the Illumina Novaseq X Plus sequencing platform (Illumina Inc., CA, United States) to generate raw reads.

#### 2.11.2 Data analysis

Clean reads were achieved by processing the raw reads to remove fitness, null, short, or low-quality sequences. The gene expression was standardized using FPKM (Fragments Per Kilobase per Million mapped fragments) in RESM software. Volcano plots were generated for the different groups based on the x-axis of log2 fold change value and y-axis of the -log10 P-value. Differential genes (DEGs) between groups were identified using DESeq2. In the analysis of DEGs, a False Discovery Rate (FDR) correction was implemented to maintain the proportion of false positives at 5% in the final results. Gene Ontology (GO) and the Kyoto Encyclopedia of Genes and Genomes (KEGG) were used for pathways enrichment analyses of DEGs.

### 2.12 Statistical analysis

Statistical analysis was performed using GraphPad Prism version 8.0 (GraphPad Software, San Diego, CA, United States). The normality of continuous data was assessed via the Shapiro-Wilk test, and homogeneity of variance was assessed via the Levene’s test. For comparisons between two groups, the independent samples t-test was employed, while one-way analysis of variance (ANOVA) was utilized for comparisons among multiple groups. The *P* values were calculated using the Least Significant Difference (LSD) test in cases of homogeneity of variance, and Welch’s t-test was applied for cases of heterogeneity of variance. Mann-Whitney test was used for data without normal distribution. A *P* value of less than 0.05 was considered statistically significant, whereas a p value of less than 0.01 indicated highly significant differences. All probability values were computed as two-tailed.

## 3 Results

### 3.1 Chemical constituents in ABS

**FIGURE 1:**
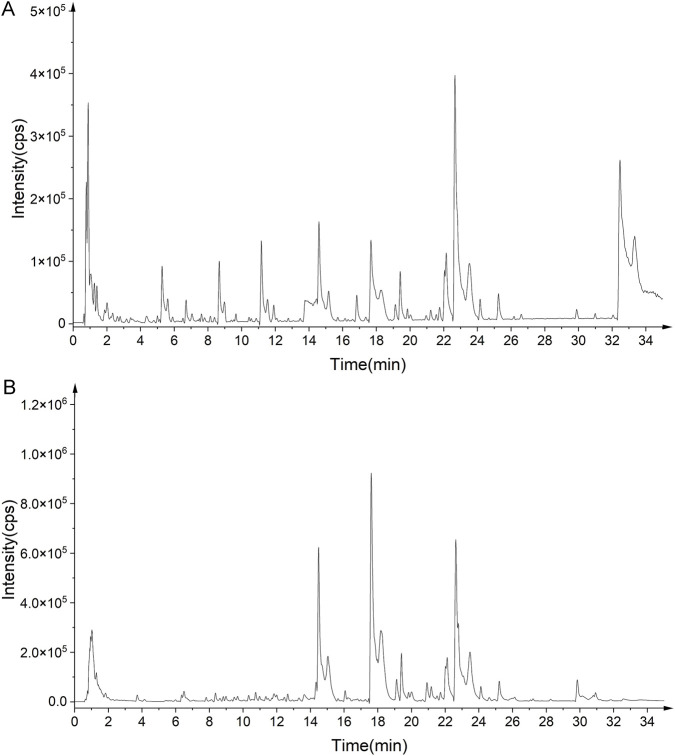
Base peak ion chromatogram of *Anshen Bunao Syrup* in positive ion mode **(A)** and negative ion mode **(B)**.

Chemical information for six material herbs was collected through literature search and database matching, including PubChem (https://pubchem.ncbi.nlm.nih.gov/) and TCMSP (https://old.tcmsp-e.com/tcmsp.php). Details of molecular weight, molecular formula, chemical makeup, and chemical structure were entered into a database. PeakView software was utilized to get details pertaining to the retention duration, precise molecular weight, and fragment ions of every molecule. By examining the fragmentation patterns of the compounds and consulting databases, the chemical composition of ABS was ascertained with less than 10 ppm molecular weight error. According to the findings, ABS is comprised of 159 different chemical components, such as phenols, flavonoids, terpenoids, amino acids, glycosides, and nucleosides (see Table 1). The base peak chromatograms for the ABS samples in both positive and negative ion modes are shown in Figure 1.

**TABLE 1:** The detail information of 159 chemical components in *Anshen Bunao Syrup*.

No.	Compound	Molecular formula	CAS number	Molecular weight	R.T. (min)	m/z	Adduct type	Error (ppm)	Source
1	Palmitic acid	C_16_H_32_O_2_	57-10-3	256.2402	0.15	257.247	[M + H]^+^	8	*Cervi Cornu Pantotrichum*
									*Zingiberis Rhizoma*
									*Fructus Jujubae*
2	Spermidine	C_7_H_19_N_3_	124-20-9	145.1579	0.66	146.165	[M + H]^+^	9.6	*Cervi Cornu Pantotrichum*
3	Arginine	C_6_H_14_N_4_O_2_	74-79-3	174.1117	0.73	175.12	[M + H]^+^	0.4	*Cervi Cornu Pantotrichum*
4	D-(+)-Glucose	C_6_H_12_O_6_	50-99-7	180.0634	0.8	179.056	[M-H]^-^	3.3	*Fructus Jujubae*
5	Proline	C_5_H_9_NO_2_	147-85-3	115.0633	0.85	116.07	[M + H]^+^	4.4	*Cervi Cornu Pantotrichum*
6	D-Sucrose	C_12_H_22_O_11_	57-50-1	342.1162	0.95	341.11	[M-H]^-^	0.1	*Fructus Jujubae*
7	Malic acid	C_4_H_6_O_5_	617-48-1	134.0215	1	133.014	[M-H]^-^	1.2	*Fructus Jujubae*
8	Niacin	C_6_H_5_NO_2_	59-67-6	123.032	1.45	124.039	[M + H]^+^	4.8	*Cervi Cornu Pantotrichum*
									*Fructus Jujubae*
9	Adenine	C_5_H_5_N_5_	73-24-5	135.0545	1.47	136.062	[M + H]^+^	0.4	*Fructus Jujubae*
10	Guanine	C_5_H_5_N_5_O	73-40-5	151.0494	1.47	152.057	[M + H]^+^	0.4	*Fructus Jujubae*
11	p-Xylene	C_8_H_10_	106-42-3	106.0782	1.55	107.085	[M + H]^+^	3.6	*Radix Glycyrrhizae*
12	Cytidine	C_9_H_13_N_3_O_5_	65-46-3	243.0855	1.6	244.093	[M + H]^+^	1.2	*Fructus Jujubae*
13	Succinic acid	C_4_H_6_O_4_	110-15-6	118.0266	2.13	117.02	[M-H]^-^	5.1	*Fructus Jujubae*
14	L-(+)-Isoleucine	C_6_H_13_NO_2_	73-32-5	131.0946	2.29	132.101	[M + H]^+^	3.6	*Cervi Cornu Pantotrichum*
15	Xanthine	C_5_H_4_N_4_O_2_	69-89-6	152.0334	2.54	153.041	[M + H]^+^	8.2	*Fructus Jujubae*
16	L-Tyrosine	C_9_H_11_NO_3_	60-18-4	181.0739	2.76	182.082	[M + H]^+^	7.5	*Cervi Cornu Pantotrichum*
17	o-Coumaric acid	C_9_H_8_O_3_	614-60-8	164.0473	2.77	165.055	[M + H]^+^	10	*Fructus Jujubae*
18	Uridine	C_9_H_12_N_2_O_6_	58-96-8	244.0695	2.97	243.062	[M-H]^-^	0.8	*Cervi Cornu Pantotrichum*
									*Fructus Jujubae*
19	Uracil	C_4_H_4_N_2_O_2_	66-22-8	112.0272	3.01	113.034	[M + H]^+^	4	*Cervi Cornu Pantotrichum*
20	o-Xylene	C_8_H_10_	95-47-6	106.0782	3.51	107.085	[M + H]^+^	1.3	*Radix Glycyrrhizae*
21	β-Terpinene	C_10_H_16_	99-84-3	136.1252	3.69	137.132	[M + H]^+^	5.1	*Zingiberis Rhizoma*
									*Radix Glycyrrhizae*
22	γ-Terpinene	C_10_H_16_	99-85-4	136.1252	3.69	137.132	[M + H]^+^	5.1	*Zingiberis Rhizoma*
									*Radix Glycyrrhizae*
23	α-Terpinene	C_10_H_16_	99-86-5	136.1252	3.69	137.132	[M + H]^+^	5.1	*Zingiberis Rhizoma*
									*Radix Glycyrrhizae*
24	Gallic acid	C_7_H_6_O_5_	149-91-7	170.0215	3.79	171.029	[M + H]^+^	6.7	*Fructus Jujubae*
25	Thymidine	C_10_H_14_N_2_O_5_	50-89-5	242.0903	4.96	241.083	[M-H]^-^	5.4	*Fructus Jujubae*
26	4-[(2-Cyclooctyn-1-yloxy)methyl]benzoic acid	C_16_H_18_O_3_	815591-73-2	258.1256	5.02	257.115	[M-H]^-^	2.8	*Radix Glycyrrhizae*
27	Adenosine	C_10_H_13_N_5_O_4_	58-61-7	267.0967	5.32	268.105	[M + H]^+^	5.9	*Fructus Jujubae*
28	m-Xylene	C_8_H_10_	108-38-3	106.0782	5.45	107.085	[M + H]^+^	0.5	*Radix Glycyrrhizae*
29	DL-Phenylalanine	C_9_H_11_NO_2_	150-30-1	165.079	5.55	166.086	[M + H]^+^	1.5	*Cervi Cornu Pantotrichum*
									*Fructus Jujubae*
30	Guanosine	C_10_H_13_N_5_O_5_	118-00-3	283.0917	5.75	284.1	[M + H]^+^	0.4	*Fructus Jujubae*
31	Inosine	C_10_H_12_N_4_O_5_	58-63-9	268.0808	5.88	267.074	[M-H]^-^	5.5	*Fructus Jujubae*
32	Polygoacetophenoside	C_14_H_18_O_10_	110906-84-8	346.09	6.01	345.082	[M-H]^-^	0.2	*Radix polygoni multiflori Preparata*
33	Adenosine cyclic phosphate	C_10_H_12_N_5_O_6_P	60-92-4	329.0525	6.11	328.046	[M-H]^-^	0.8	*Fructus Jujubae*
34	cGMP	C_10_H_12_N_5_O_7_P	7665-99-8	345.0474	6.11	344.04	[M-H]^-^	7.4	*Fructus Jujubae*
35	3-Hydroxyglabrol	C_25_H_28_O_5_	74148-41-7	408.1937	6.53	409.196	[M + H]^+^	7.2	*Radix Glycyrrhizae*
36	Catechol	C_6_H_6_O_2_	120-80-9	110.0368	6.69	109.03	[M-H]^-^	7.5	*Fructus Jujubae*
37	Protocatechuic acid	C_7_H_6_O_4_	99-50-3	154.0266	7	155.034	[M + H]^+^	5.6	*Fructus Jujubae*
									*Radix Glycyrrhizae*
38	Maltol	C_6_H_6_O_3_	118-71-8	126.0317	7.04	127.039	[M + H]^+^	3.6	*Epimedii Folium*
39	Salidroside	C_14_H_20_O_7_	10338-51-9	300.1209	8.28	301.127	[M + H]^+^	3.3	*Epimedii Folium*
40	Benzaldehyde	C_7_H_6_O	100-52-7	106.0419	9.03	107.049	[M + H]^+^	1.5	*Fructus Jujubae*
41	(S)-(−)-Stepholidine	C_19_H_21_NO_4_	16562-13-3	327.147	9.57	328.155	[M + H]^+^	1.2	*Fructus Jujubae*
42	Vanillic acid	C_8_H_8_O_4_	121-34-6	168.0422	9.97	169.049	[M + H]^+^	3.6	*Fructus Jujubae*
43	Moupinamide	C_18_H_19_NO_4_	66648-43-9	313.1314	10.2	314.139	[M + H]^+^	0.9	*Radix polygoni multiflori Preparata*
44	Chlorogenic acid	C_16_H_18_O_9_	327-97-9	354.0951	10.42	355.103	[M + H]^+^	3.2	*Fructus Jujubae*
45	Protopine	C_20_H_19_NO_5_	130-86-9	353.1263	10.55	354.134	[M + H]^+^	3.2	*Fructus Jujubae*
46	Caffeic acid	C_9_H_8_O_4_	331-39-5	180.0422	10.62	179.035	[M-H]^-^	5.7	*Fructus Jujubae*
47	Vomifoliol	C_13_H_20_O_3_	23526-45-6	224.1412	10.63	225.149	[M + H]^+^	4.2	*Fructus Jujubae*
48	Narcissin	C_28_H_32_O_16_	604-80-8	624.169	10.71	623.164	[M-H]^-^	1	*Radix Glycyrrhizae*
49	Magnoflorine	C_20_H_24_NO_4_+	2141/9/5	342.1705	11.14	342.171	[M + H]^+^	3.9	*Fructus Jujubae*
50	Syringic acid	C_9_H_10_O_5_	530-57-4	198.0528	11.51	199.06	[M + H]^+^	1.3	*Fructus Jujubae*
51	Olivil	C_20_H_24_O_7_	2955-23-9	376.1522	11.73	375.147	[M-H]^-^	0.6	*Epimedii Folium*
52	Licocoumarone	C_20_H_20_O_5_	118524-14-4	340.1311	11.81	341.139	[M + H]^+^	1	*Radix Glycyrrhizae*
53	Glepidotin B	C_20_H_20_O_5_	87440-56-0	340.1311	11.82	341.139	[M + H]^+^	2.1	*Radix Glycyrrhizae*
54	Zingerone	C_11_H_14_O_3_	122-48-5	194.0943	11.96	195.101	[M + H]^+^	1.6	*Zingiberis Rhizoma*
55	Roseoside I	C_19_H_30_O_8_	54835-70-0	386.1941	11.98	387.202	[M + H]^+^	1.8	*Fructus Jujubae*
56	Licoflavone A	C_20_H_18_O_4_	61153-77-3	322.1205	12.24	323.127	[M + H]^+^	3.2	*Radix Glycyrrhizae*
57	Rhein	C_15_H_8_O_6_	478-43-3	284.0321	12.32	283.029	[M-H]^-^	1.9	*Radix polygoni multiflori Preparata*
58	Morachalcone A	C_20_H_20_O_5_	76472-88-3	340.1311	12.32	341.139	[M + H]^+^	1.3	*Radix Glycyrrhizae*
59	Caohuoside D	C_28_H_34_O_12_	161504-79-6	562.205	12.34	563.209	[M + H]^+^	2.3	*Epimedii Folium*
60	(−)-Riboflavin	C_17_H_20_N_4_O_6_	83-88-5	376.1383	12.9	377.145	[M + H]^+^	0.7	*Fructus Jujubae*
61	Licoricone	C_22_H_22_O_6_	51847-92-8	382.1416	12.98	381.119	[M-H]^-^	6.6	*Radix Glycyrrhizae*
62	Nicotiflorin	C_27_H_30_O_15_	17650-84-9	594.1585	13.04	595.165	[M + H]^+^	2.6	*Radix Glycyrrhizae*
63	Scopoletin	C_10_H_8_O_4_	92-61-5	192.0422	13.13	193.05	[M + H]^+^	4	*Fructus Jujubae*
									*Radix Glycyrrhizae*
64	4′,7-Dihydroxyflavone	C_15_H_10_O_4_	2196-14-7	254.0579	13.18	253.052	[M-H]^-^	8.1	*Radix Glycyrrhizae*
65	Puerarin	C_21_H_20_O_9_	3681-99-0	416.1107	13.3	417.119	[M + H]^+^	5.3	*Fructus Jujubae*
66	Valine	C_5_H_11_NO_2_	72-18-4	117.079	13.57	118.086	[M + H]^+^	2.6	*Cervi Cornu Pantotrichum*
67	Naringin	C_27_H_32_O_14_	10236-47-2	580.1792	13.62	581.187	[M + H]^+^	8.1	*Radix Glycyrrhizae*
68	4-Hydroxybenzaldehyde	C_7_H_6_O_2_	123-08-0	122.0368	13.81	123.044	[M + H]^+^	3.8	*Cervi Cornu Pantotrichum*
69	Naringenin	C_15_H_12_O_5_	480-41-1	272.0685	14.03	273.076	[M + H]^+^	0.2	*Radix Glycyrrhizae*
70	Vicenin 2	C_27_H_30_O_15_	23666-13-9	594.1585	14.1	595.167	[M + H]^+^	4.7	*Fructus Jujubae*
									*Radix Glycyrrhizae*
71	Isoliquiritigenin	C_15_H_12_O_4_	961-29-5	256.0736	14.43	257.081	[M + H]^+^	3.1	*Epimedii Folium*
									*Radix Glycyrrhizae*
72	Swertisin	C_22_H_22_O_10_	6991/10/2	446.1213	14.47	447.128	[M + H]^+^	7.9	*Fructus Jujubae*
73	Brevicornin	C_22_H_24_O_7_	173792-49-9	400.1522	14.5	401.157	[M + H]^+^	6.5	*Epimedii Folium*
74	Emodin anthrone	​	491-60-1	256.0735	14.52	257.081	[M + H]^+^	1	*Radix polygoni multiflori Preparata*
		C_15_H_12_O_4_							
75	Pinocembrin	C_15_H_12_O_4_	480-39-7	256.0736	14.72	257.081	[M + H]^+^	3.3	*Radix Glycyrrhizae*
76	Rutin	C_27_H_30_O_16_	153-18-4	610.1534	14.91	611.162	[M + H]^+^	2.1	*Fructus Jujubae, Radix Glycyrrhizae*
77	Isovitexin	C_21_H_20_O_10_	29702-25-8	432.1056	14.98	433.115	[M + H]^+^	5.5	*Fructus Jujubae*
78	Vitexin	C_21_H_20_O_10_	3681-93-4	432.1056	15.03	433.114	[M + H]^+^	2.6	*Fructus Jujubae*
79	Cycloolivil	C_20_H_24_O_7_	3064/5/9	376.1522	15.27	377.157	[M + H]^+^	3.2	*Epimedii Folium*
80	Shinpterocarpin	C_20_H_18_O_4_	157414-04-5	322.1205	15.69	323.129	[M + H]^+^	3.3	*Radix Glycyrrhizae*
81	Glycyrin	C_22_H_22_O_6_	66056-18-6	382.1416	15.7	383.15	[M + H]^+^	0	*Radix Glycyrrhizae*
82	Trifolin	C_21_H_20_O_11_	23627-87-4	448.1006	15.91	449.11	[M + H]^+^	2.3	*Epimedii Folium*
83	Robinetin	C_15_H_10_O_7_	490-31-3	302.0426	16.14	303.051	[M + H]^+^	2.4	*Epimedii Folium*
84	Hyperoside	C_21_H_20_O_12_	482-36-0	464.0955	16.14	465.104	[M + H]^+^	3	*Epimedii Folium*
									*Fructus Jujubae*
85	Piceid	C_20_H_22_O_8_	27208-80-6	390.1315	16.17	391.137	[M + H]^+^	0.6	*Radix polygoni multiflori Preparata*
86	Isoquercetin	C_21_H_20_O_12_	482-35-9	464.0955	16.26	465.101	[M + H]^+^	2	*Epimedii Folium*
									*Fructus Jujubae*
									*Radix Glycyrrhizae*
87	Spinosin	C_28_H_32_O_15_	72063-39-9	608.1741	16.37	609.182	[M + H]^+^	8	*Fructus Jujubae*
88	Astragalin	C_21_H_20_O_11_	480-10-4	448.1006	16.38	449.107	[M + H]^+^	2	*Epimedii Folium*
									*Radix Glycyrrhizae*
89	Icariside A7	C_23_H_26_O_10_	1177924-62-7	462.1526	16.67	461.147	[M-H]^-^	8.7	*Epimedii Folium*
90	Chryseriol	C_16_H_12_O_6_	491-71-4	300.0634	16.71	299.056	[M-H]^-^	3.4	*Epimedii Folium*
									*Fructus Jujubae*
91	Neochanin	C_16_H_12_O_4_	485-72-3	268.0736	17.29	269.082	[M + H]^+^	1.7	*Radix Glycyrrhizae*
92	Kaempferitrin	C_27_H_30_O_14_	482-38-2	578.1636	17.54	579.172	[M + H]^+^	2.4	*Epimedii Folium*
93	4-Hydroxybenzoic acid	C_7_H_6_O_3_	99-96-7	138.0317	17.64	139.039	[M + H]^+^	0.3	*Cervi Cornu Pantotrichum*
94	Hexandraside E	C_32_H_38_O_16_	139955-75-2	678.216	18.11	679.223	[M + H]^+^	3.1	*Epimedii Folium*
95	Chrysophanic acid	C_15_H_10_O_4_	481-74-3	254.0579	18.4	255.066	[M + H]^+^	3.1	*Radix polygoni multiflori Preparata*
96	Ikarisoside A	C_26_H_28_O_10_	55395-07-8	500.1682	18.53	501.174	[M + H]^+^	2.1	*Epimedii Folium*
97	Bolusanthin III	C_16_H_14_O_4_	68178-63-2	270.0892	18.55	271.097	[M + H]^+^	8.7	*Radix Glycyrrhizae*
98	Diflucortolone valerate	C_27_H_36_F_2_O_5_	59198-70-8	478.2531	18.75	477.237	[M-H]^-^	6.9	*Epimedii Folium*
									*Fructus Jujubae*
99	Citreorosein	C_15_H_10_O_6_	481-73-2	286.0477	18.9	287.054	[M + H]^+^	1.1	*Radix polygoni multiflori Preparata*
100	(+)-[6]^-^Gingerol	C_17_H_26_O_4_	23513-14-6	294.1831	18.95	295.187	[M + H]^+^	1.4	*Zingiberis Rhizoma*
101	Rouhuoside	C_38_H_48_O_20_	131862-37-8	824.2739	19.08	823.273	[M-H]^-^	8.3	*Epimedii Folium*
102	Licoisoflavone A	C_20_H_18_O_6_	66056-19-7	354.1103	19.11	355.116	[M + H]^+^	3.3	*Radix Glycyrrhizae*
103	Epimedoside D	C_37_H_46_O_19_	39049-18-8	794.2633	19.16	795.27	[M + H]^+^	3.5	*Epimedii Folium*
104	Baohuoside I	C_27_H_30_O_10_	113558-15-9	514.1839	19.68	515.189	[M + H]^+^	2.6	*Epimedii Folium*
105	Epimedoside E	C_37_H_46_O_19_	39049-19-9	794.2633	19.73	795.271	[M + H]^+^	5.5	*Epimedii Folium*
106	Acuminatoside	C_45_H_60_O_24_	142735-71-5	984.3474	20.34	983.344	[M-H]^-^	8.3	*Epimedii Folium*
107	Icariside C1	C_21_H_38_O_8_	108906-50-9	418.2567	20.81	417.251	[M-H]^-^	5.4	*Epimedii Folium*
108	Apigenin	C_15_H_10_O_5_	520-36-5	270.0528	20.93	271.06	[M + H]^+^	1.6	*Epimedii Folium*
109	L-(+)-Catechin	C_15_H_14_O_6_	18829-70-4	290.079	21.12	289.072	[M-H]^-^	2.7	*Fructus Jujubae*
110	(−)-Epicatechin	C_15_H_14_O_6_	490-46-0	290.079	21.15	289.071	[M-H]^-^	1.7	*Fructus Jujubae*
111	(+)-Epicatechin	C_15_H_14_O_6_	35323-91-2	290.079	21.2	289.071	[M-H]^-^	1.8	*Fructus Jujubae*
112	D-(+)-Catechin	C_15_H_14_O_6_	154-23-4	290.079	21.29	289.071	[M-H]^-^	2.3	*Fructus Jujubae*
113	Butylated hydroxytoluene	C_15_H_24_O	128-37-0	220.1827	21.6	221.19	[M + H]^+^	0.4	*Radix Glycyrrhizae*
114	Epimedin A1	C_39_H_50_O_20_	140147-77-9	838.2895	21.73	839.298	[M + H]^+^	1.9	*Epimedii Folium*
115	Paradol	C_17_H_26_O_3_	27113-22-0	278.1882	21.8	279.194	[M + H]^+^	1.8	*Zingiberis Rhizoma*
116	Epimedoside C	C_26_H_28_O_11_	55394-98-4	516.1632	21.81	517.172	[M + H]^+^	3.5	*Epimedii Folium*
117	Archin	C_15_H_10_O_5_	518-82-1	270.0528	21.89	271.06	[M + H]^+^	3.1	*Radix polygoni multiflori Preparata, Epimedii Folium*
118	Wushanicariin	C_27_H_30_O_11_	115516-53-5	530.1788	22.11	531.185	[M + H]^+^	0.2	*Epimedii Folium*
119	Shogaol	C_17_H_24_O_3_	555-66-8	276.1725	22.52	277.178	[M + H]^+^	4.6	*Zingiberis Rhizoma*
120	SYSU-00655	C_21_H_20_O_6_	​	368.12	22.59	369.133	[M + H]^+^	0.1	*Epimedii Folium*
121	Icariin A	C_33_H_40_O_15_	118525-35-2	676.2367	22.68	677.244	[M + H]^+^	2.8	*Epimedii Folium*
122	Epmedin C	C_39_H_50_O_19_	​	822.2946	22.8	823.302	[M + H]^+^	2.7	*Epimedii Folium*
123	Epimedin B	C_38_H_48_O_19_	110623-73-9	808.279	22.92	809.286	[M + H]^+^	2.9	*Epimedii Folium*
124	Questin	C_16_H_12_O_5_	3774-64-9	284.0685	23.15	283.064	[M-H]^-^	7.4	*Radix polygoni multiflori Preparata*
125	Parietin	C_16_H_12_O_5_	521-61-9	284.0685	23.15	283.064	[M-H]^-^	7.4	*Radix polygoni multiflori Preparata*
126	(−)-Maackiain	C_16_H_12_O_5_	2035-15-6	284.0685	23.22	285.076	[M + H]^+^	4.9	*Radix Glycyrrhizae*
127	Luteolin	C_15_H_10_O_6_	491-70-3	286.0477	23.31	285.039	[M-H]^-^	0.2	*Epimedii Folium*
									*Radix Glycyrrhizae*
128	Kaempferol	C_15_H_10_O_6_	520-18-3	286.0477	23.31	285.039	[M-H]^-^	0.2	*Epimedii Folium*
									*Radix Glycyrrhizae*
129	Wanepimedoside A	C_33_H_42_O_15_	181939-44-6	678.2524	25.06	679.259	[M + H]^+^	1.8	*Epimedii Folium*
130	Bilobanol	C_15_H_22_O_2_	959582-37-7	234.162	25.76	235.169	[M + H]^+^	3.4	*Epimedii Folium*
131	Caohuoside B	C_45_H_56_O_23_	159650-19-8	964.3212	26.16	965.331	[M + H]^+^	3.5	*Epimedii Folium*
132	Licoflavonol	C_20_H_18_O_6_	60197-60-6	354.1103	26.32	355.12	[M + H]^+^	2.6	*Radix Glycyrrhizae*
133	Calycosin	C_16_H_12_O_5_	20575-57-9	284.0685	26.51	283.061	[M-H]^-^	0.7	*Radix Glycyrrhizae*
134	Prunetin	C_16_H_12_O_5_	552-59-0	284.0685	26.51	283.061	[M-H]^-^	0.7	*Radix Glycyrrhizae*
135	Gancaonin D	C_21_H_20_O_7_	124596-88-9	384.1209	26.72	385.129	[M + H]^+^	3.3	*Radix Glycyrrhizae*
136	Desmethylicaritin	C_20_H_18_O_6_	28610-31-3	354.1103	26.74	355.117	[M + H]^+^	2.3	*Epimedii Folium*
137	Ikarisoside F	C_31_H_36_O_14_	113558-14-8	632.2105	26.76	633.219	[M + H]^+^	3.9	*Epimedii Folium*
138	Yinyanghuo C	C_20_H_16_O_5_	149182-47-8	336.0998	27.08	337.105	[M + H]^+^	3.7	*Epimedii Folium*
139	Glyasperin F	C_20_H_18_O_6_	145382-61-2	354.1103	27.08	355.118	[M + H]^+^	1.2	*Radix Glycyrrhizae*
140	Licoisoflavanone	C_20_H_18_O_6_	66067-26-3	354.1103	27.11	355.117	[M + H]^+^	7.3	*Radix Glycyrrhizae*
141	6-Gingediacetate	C_21_H_32_O_6_	143615-75-2	380.2199	27.51	381.226	[M + H]^+^	5.5	*Zingiberis Rhizoma*
142	Icariside I	C_27_H_30_O_11_	56725-99-6	530.1788	27.8	531.187	[M + H]^+^	8.8	*Epimedii Folium*
143	Chrysarobin	C_15_H_12_O_3_	491-58-7	240.0786	28.99	239.069	[M-H]^-^	7.6	*Radix polygoni multiflori Preparata*
144	Xanthorrhizol	C_15_H_22_O	30199-26-9	218.1671	29.05	219.174	[M + H]^+^	1.5	*Zingiberis Rhizoma*
145	Glycyrrhizin	C_42_H_62_O_16_	1405-86-3	822.4038	29.86	823.412	[M + H]^+^	5.3	*Radix Glycyrrhizae*
146	Enoxolone	C_30_H_46_O_4_	471-53-4	470.3396	29.93	471.345	[M + H]^+^	0.2	*Radix Glycyrrhizae*
147	Zizyberanalic acid	C_30_H_46_O_4_	67594-73-4	470.3396	29.95	471.346	[M + H]^+^	0.1	*Fructus Jujubae*
148	Icariin	C_33_H_40_O_15_	489-32-7	676.2367	30.03	677.245	[M + H]^+^	8.7	*Epimedii Folium*
149	Icaritin	C_21_H_20_O_6_	118525-40-9	368.126	30.46	369.134	[M + H]^+^	7.1	*Epimedii Folium*
150	Gingerglycolipid A	C_33_H_56_O_14_	145937-22-0	676.367	30.47	675.361	[M-H]^-^	6.6	*Zingiberis Rhizoma*
151	Gancaonin B	C_21_H_20_O_6_	124596-86-7	368.126	30.48	369.13	[M + H]^+^	2.1	*Radix Glycyrrhizae*
152	Glycycoumarin	C_21_H_20_O_6_	94805-82-0	368.126	30.95	369.134	[M + H]^+^	4.7	*Radix Glycyrrhizae*
153	Sagittatosdie B	C_32_H_38_O_14_	118525-36-3	646.2262	31.45	647.235	[M + H]^+^	1.1	*Epimedii Folium*
154	Dibutyl phthalate	C_16_H_22_O_4_	84-74-2	278.1518	32.48	279.159	[M + H]^+^	8.9	*Zingiberis Rhizoma*
155	Epimedoside	C_37_H_44_O_17_	106441-31-0	760.2578	32.84	761.267	[M + H]^+^	1.2	*Epimedii Folium*
156	1-Monolinolein	C_21_H_38_O_4_	2277-28-3	354.277	33.4	355.288	[M + H]^+^	6.9	*Zingiberis Rhizoma*
157	10-Gingerol	C_21_H_34_O_4_	23513-15-7	350.2457	33.91	351.247	[M + H]^+^	10	*Zingiberis Rhizoma*
158	10-Gingerdione	C_21_H_32_O_4_	79067-90-6	348.2301	34.27	349.236	[M + H]^+^	4.7	*Zingiberis Rhizoma*
159	Isovaleraldehyde	C_5_H_10_O	590-86-3	86.0732	34.32	87.0785	[M + H]^+^	2	*Zingiberis Rhizoma*

R.T., retention time.

### 3.2 Effects of ABS

**FIGURE 2:**
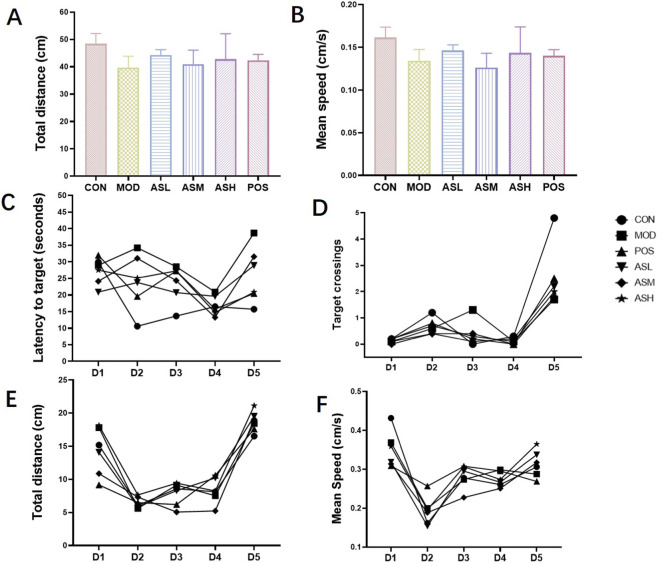
Results of behavior tests. Total distance **(A)** and Mean speed **(B)** in open field test. Latency to target **(C)**, Target crossing **(D)**, Total distance **(E)** and Mean speed **(F)** in Morris water maze test. CON, healthy control group; MOD, aging model group; ASL, *Anshen Bunao Syrup* low-dose administration group; ASM, *Anshen Bunao Syrup* medium-dose administration group; ASH, *Anshen Bunao Syrup* high-dose administration group; POS, positive control group.

Open-field test is used to evaluate rodent experimental animals’ voluntary movement behavior, exploratory behavior and tension. As shown in Figures 2A,B, ABS increased locomotor activity more than rapamycin. Spatial learning was tested daily (4 trials/day) using the MWM on days 53–58. The aging rats required a significantly longer time to find the platform compared to control rats (*P* = 0.0363) using Welch’s t-test. They also showed markedly fewer crossing times of the target platform compared to control rats (*P* = 0.0245). In contrast, ABS treatment shortened the latency to the target and raised the number of crossing the target (Figures 2C,D), while also tending to elevate the total distance and mean speed in MWM (Figures 2E,F). For spatial and learning memory, the therapeutic effect of the high-dose ABS was comparable to rapamycin. These results suggested that ABS treatment improved aging rats’ locomotor activity and spatial and learning memory.

**FIGURE 3:**
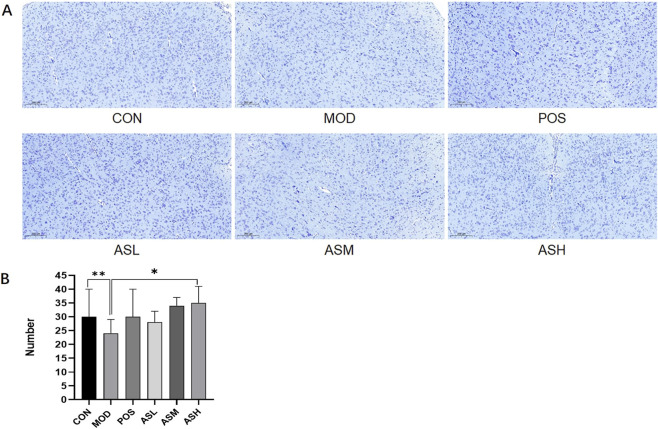
Hematoxylin and eosin (HE) staining of rats’ skin (×100). **(A)** HE staining photomicrographs from different groups. **(B)** Cortical thickness in the field of view as bar graphs. CON, healthy control group; MOD, aging model group; POS, positive control group; ASL, *Anshen Bunao Syrup* low-dose administration group; ASM, *Anshen Bunao Syrup* medium-dose administration group; ASH, *Anshen Bunao Syrup* high-dose administration group.

**FIGURE 4:**
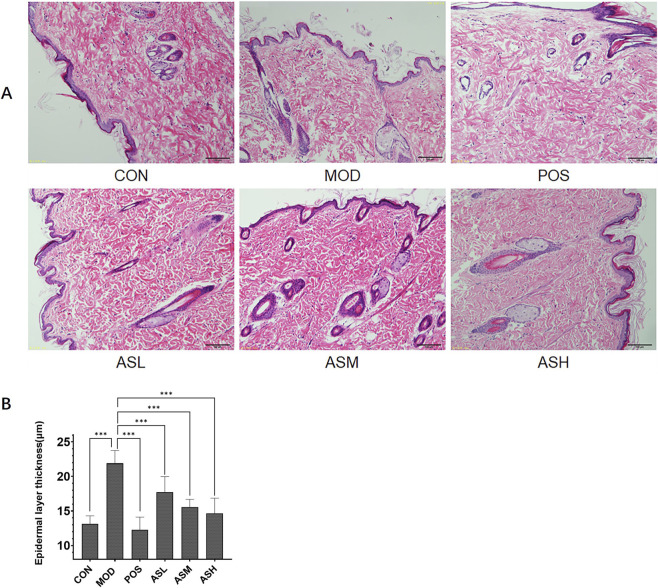
Brains of rats stained with Nissl (×100). **(A)** Nissl staining photomicrographs from different groups. **(B)** Neurons in the field of view as bar graphs. CON, healthy control group; MOD, aging model group; POS, positive control group; ASL, *Anshen Bunao Syrup* low-dose administration group; ASM, *Anshen Bunao Syrup* medium-dose administration group; ASH, *Anshen Bunao Syrup* high-dose administration group.

The condition of the skin is an essential indicator of aging. H&E staining of neck skin indicated that aging rats (MOD) exhibited significantly increased epidermal thickness (*P* < 0.001), distorted elastic fibers, and irregularly arranged epidermal cells. At the same time, in the ABS-treated groups, these pathological changes were notably ameliorated (Figure 3). Previous studies showed that the number of neurons in the brain decreases with aging, and Nissl staining of brain tissue was conducted to evaluate neuronal survival. Results displayed that aging rats had a significantly lower cortical neuron density compared with the control group (*P* < 0.05). Rats receiving ABS had more neurons than aging rats, demonstrating that receiving ABS prevented aging-induced neuronal injuries (Figure 4).

**FIGURE 5:**
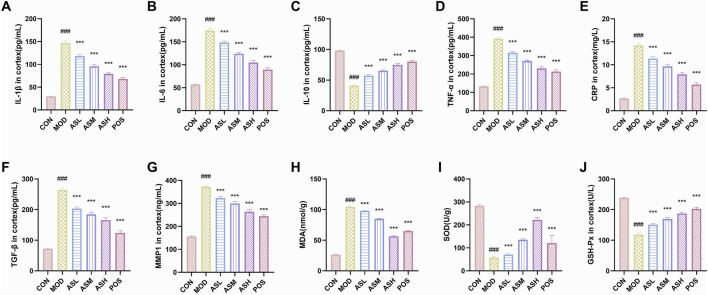
Enzyme-linked immunosorbent assay analysis of senescence-associated secretory phenotype. **(A)** Interleukin-1β (IL-1β). **(B)** Interleukin-6 (IL-6). **(C)** Interleukin-10 (IL-10). **(D)** Tumor necrosis factor-alpha (TNF-α). **(E)** C-reactive protein (CRP). **(F)** Transforming growth factor beta (TGF-β). **(G)** Matrix metallopeptidase 1 (MMP1). **(H)** Malondialdehyde (MDA). **(I)** Superoxide dismutase (SOD). **(J)** Glutathione peroxidase (GSH-Px). CON, healthy control group (n = 10); MOD, aging model group (n = 10); ASL, *Anshen Bunao Syrup* low-dose administration group (n = 10); ASM, *Anshen Bunao Syrup* medium-dose administration group (n = 10); ASH, *Anshen Bunao Syrup* high-dose administration group (n = 10); POS, positive control group (n = 10). Pound signs (###*P* < 0.0001) indicated significant differences from the CON group, and asterisks (***P < 0.0001) indicated significant differences from the MOD group.

Senescent cells secrete various biologically active molecules collectively known as the SASP. The factors include growth factors, inflammatory factors, metalloproteinases, and reactive oxygen species. Thus, we examined the content of inflammatory factors IL-1β, IL-6, IL-10, TNF-α, CRP, growth factors TGF-β, metalloproteinase MMP-1, and oxidative stress-related indicators MDA, SOD, GSH-Px in cortex of each rats using ELISA kits. Data showed that aging upregulates the levels of pro-inflammatory factors, including IL-1β, IL-6, TNF-α, and CRP, while downregulate the level of anti-inflammatory factor of IL-10 (*P* < 0.001, see Figures 5A–E). The content of TGF-β, and MMP-1 were upregulated in aging rats, and were downregulated by ABS treatment (*P* < 0.001, Figures 5F,G). Elevated MDA, decreased SOD and GSH-Px suggested excessive oxidative stress in aging rats (*P* < 0.001), which indicates that oxidative stress is inhibited by ABS intervention (*P* < 0.001, Figures 5H–J).

### 3.3 Mechanism of ABS against aging

**FIGURE 6:**
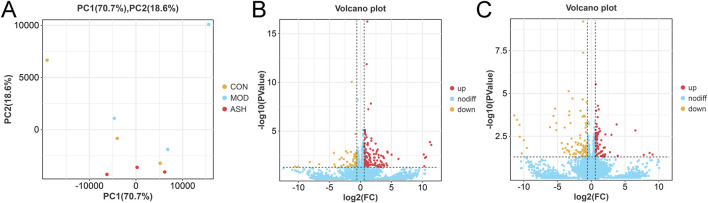
Results of differential genes expression analysis. **(A)** Score plot of principal component analysis. **(B)** Volcano plot of the healthy control group compared with the aging model group. **(C)** Volcano plot of the aging model group compared with the *Anshen Bunao Syrup* high-dose administration group. Upregulated genes are marked in red, downregulated genes are marked in yellow.

**FIGURE 7:**
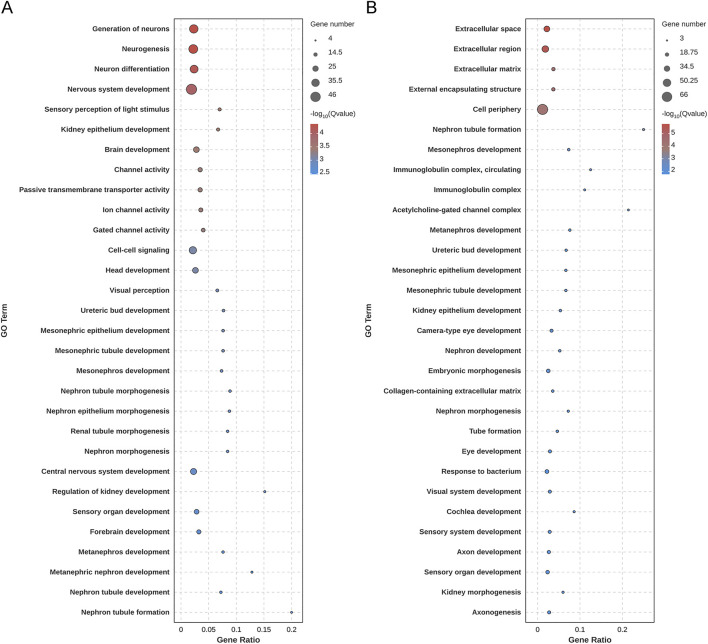
Results of GO enrichment analysis. **(A)** Bubble diagram of GO analysis based on differentially expressed genes between the healthy control group and the aging model group. **(B)** Bubble diagram of GO analysis based on differentially expressed genes between the aging model group and the *Anshen Bunao Syrup* high-dose administration group. The closer the color is to red, the smaller the Q value, indicating the greater significance of the GO term. The larger the circle, the greater the number of enriched genes.

**FIGURE 8:**
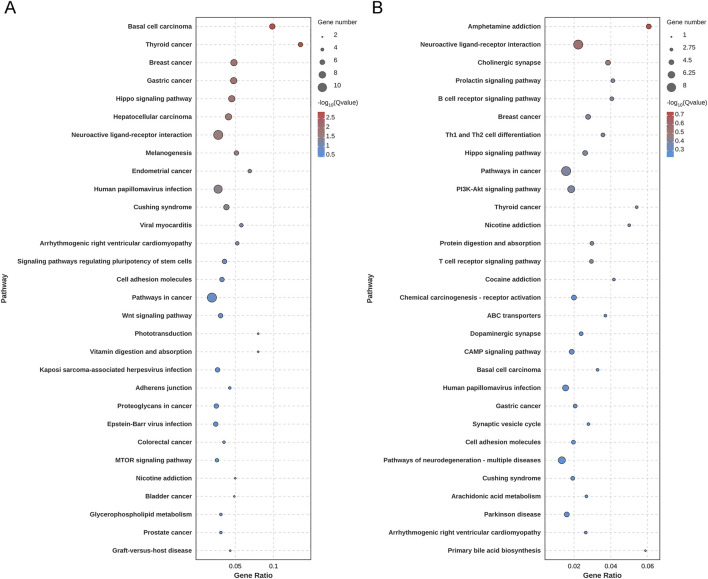
Results of KEGG enrichment analysis. **(A)** Bubble diagram of KEGG analysis based on differentially expressed genes between the healthy control group and the aging model group. **(B)** Bubble diagram of KEGG analysis based on differentially expressed genes between the aging model group and the *Anshen Bunao Syrup* high-dose administration group. The closer the color is to red, the smaller the Q value, indicating the greater significance of the pathway. The larger the circle, the greater the number of enriched genes.

In transcriptomics analysis, the score plot of principal component analysis based on differential gene expression is shown in Figure 6A. Based on *P* value and log2(FC) value, 142 upregulated genes and 63 downregulated genes in aging rats (Figure 6B) were highlighted via volcano plot. The administered with ABS caused the upregulation of 80 genes and the downregulation of 94 genes in cortex compared to the gene expression levels in the MOD group (Figure 6C), and ABS administration reversed the expression content of 281 DEGs (Supplementary Table S1). GO enrichment analysis highlighted 205 DEGs induced by D-galactose, and 174 DEGs were enriched in extracellular space, extracellular region, extracellular matrix; and moreover, genes were mostly enriched in nephron tubule formation, mesonephros development, metanephros development (Figure 7). KEGG enrichment analysis showed that the main pathways contributed to aging were hippo signaling pathway, neuroactive ligand-receptor interaction, cell adhesion molecules, signaling pathways regulating pluripotency of stem cells, Wnt signaling pathway (Figure 8A). While ABS administration mainly affected signal transduction involved in nervous system and immune system, such as neuroactive ligand-receptor interaction, prolactin signaling pathway, Th1 and Th2 cell differentiation, and arachidonic acid metabolism (Figure 8B).

**FIGURE 9:**
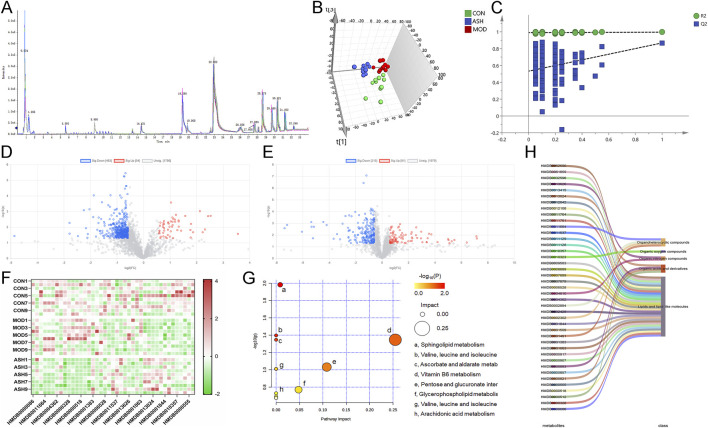
Results of metabolomic analysis. **(A)** Base peak ion chromatogram of quality control sample. **(B)** Score plot of partial least squares discriminant analysis (PLS-DA). **(C)** Permutation validation of PLS-DA. **(D)** Volcano plot of the healthy control (CON) group compared with the aging model (MOD) group. Upregulated genes are marked in red, downregulated genes are marked in blue. **(E)** Volcano plot of the MOD group compared with the *Anshen Bunao Syrup* high-dose administration (ASH) group. Upregulated genes are marked in red, downregulated genes are marked in blue. **(F)** Heat map of differential metabolite contents in different groups. The closer the color is to red, the higher the metabolite content. The closer the color is to green, the lower the metabolite content. **(G)** Bubble map of KEGG pathway analysis. The closer the color is to red, the smaller the *P* value; the larger the circle, the larger the impact value of the pathway. **(H)** Sankey diagram of metabolites classification.

As for UPLC-Q/TOF MS based metabolomics, QC samples were used to validate the stability and repeatability of the metabolomics analysis. As shown in Figure 9A, all the QC samples were tightly overlapped in the base peak chromatogram. UPLC-Q/TOF MS data showed that 5,355 features were obtained in positive mode. To further get a direct overview of the differences in global metabolic profiles among control rats, aging rats, and rats with ABS high-dose administration, the supervised PLS-DA model was conducted (Figure 9B). The permutations plot of PLS-DA indicated that the original model is valid (Figure 9C). In addition, we calculated the t-test and FC on each feature. Based on *P* value and log2(FC) value, a volcano plot of features in CON group and MOD group and a volcano plot of features in ASH group and MOD group were drawn. The characteristic metabolites of ABS in the treatment of aging were discovered according to the *P* value <0.05 and FC > 1.5 (Figures 9D,E). There were 517 metabolites expressed differentially in control and aging rats (54 upregulated and 463 downregulated). Compared with aging rats, the ABS treatment resulted in 306 metabolites changed (91 upregulated and 215 downregulated). A total of 41 differential endogenous metabolites whose contents could be reversed by ABS were identified.

17 upregulated metabolites in the MOD group compared with the CON group were decreased after treatment with ABS, and 24 downregulated metabolites were increased after administration with ABS. The differential metabolite dataset was imported into GraphPad Prism software (version 8.0) to generate a heatmap (Figure 9F). Metabolic pathway analysis was conducted with MetaboAnalyst 5.0 to explore further the pathogenesis of AD and the possible mechanism by which ABS treatment ameliorates this disease. The 41 differential endogenous metabolites were mainly involved in sphingolipid metabolism, valine, leucine and isoleucine biosynthesis, ascorbate and aldarate metabolism, vitamin B6 metabolism, arachidonic acid metabolism (Figure 9G). Figure 9H further shows that 63.83% of the differential metabolites were lipids and lipid-like molecules, indicative of significant changes in the levels of lipid metabolites in the cerebral cortices of aging rats and ABS intervention.

**FIGURE 10:**
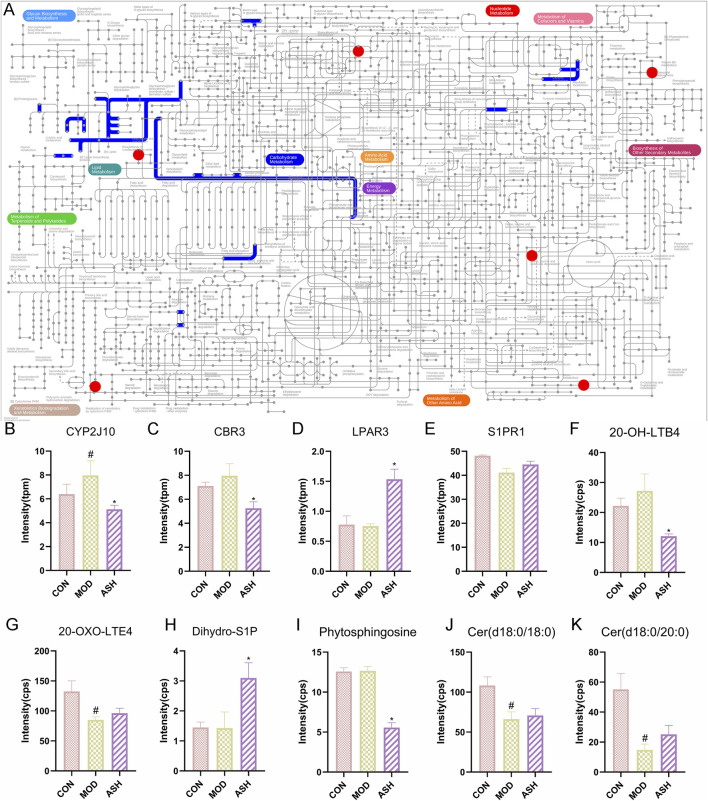
Results of integrated transcriptome and metabolome analysis. **(A)** KEGG general metabolic pathway map in iPath 3.0 for visualizing differentially expressed genes and differential metabolites. Statistical graph of CYP2J10 **(B)**, CBR3 **(C)**, LPAR3 **(D)**, S1PR1 **(E)**, 20-hydroxy-leukotriene B4 (20-OH-LTB4) **(F)**, 20-oxo-leukotriene E4 (20-OXO-LTE4) **(G)**, sphinganine 1-phosphate (dihydro-S1P) **(H)**, phytosphingosine **(I)**, Cer(d18:0/18:0) **(J)**, Cer(d18:0/20:0) **(K)**.

An in-depth analysis was conducted by integrating differential metabolites and DEGs reversed by ABS intervention using iPath 3.0, with the aim of further exploring the altered metabolic pathways associated with the anti-aging effects of ABS. The primary focus of the altered metabolic pathways was lipid metabolism. Based on the integrated transcriptomic and metabolomic analyses, it is hypothesized that ABS may exert its anti-aging effects by modulating arachidonic acid metabolism and sphingolipid metabolism (Figure 10A). To substantiate this hypothesis, a comprehensive analysis was undertaken of the key DEGs and metabolites involved in arachidonic acid metabolism (including CYP2J10, CBR3, 20-hydroxy-leukotriene B4, and 20-oxo-leukotriene E4) and sphingolipid metabolism (including LPAR3, sphinganine 1-phosphate, phytosphingosine, Cer(d18:0/18:0), and Cer(d18:0/20:0)).

The mRNA expression levels of genes modulating oxidoreductase reactions (CYP2J10, CBR3) were increased in MOD group relative to CON, and downregulated by ABS treatment (Figures 10B,C). ABS markedly reduced content of 20-hydroxy-leukotriene B4 (20-OH-LTB4) relative to MOD group (Figure 10F). However, 20-oxo-leukotriene E4 (20-oxo-LTE4) level was slightly rose in ASH and CON groups compared to MOD group (Figure 10G). The expression of LPAR3 was markedly elevated in ASH group than MOD group (Figure 10D). Compared to MOD group, ABS upregulated levels of sphinganine 1-phosphate (dihydro-S1P) and Cers, however, ABS downregulated the amount of phytosphingosine (Figures 10H–K). Additionally, we found that the expression of S1PR, one of S1P receptors, was gently upregulated by ABS (Figure 10E).

## 4 Discussion

This study showed that ABS could not only enhance learning ability, improve skin elasticity, reduce neuronal loss, and modulate SASP, but also inhibit oxidative stress and inflammation in premature aging rats. Evidences show that ageing is a major risk factor for neurodegenerative diseases (Hou et al., 2019). As this elderly population increases, the financial burden of age-related health disorders is increasing, and effective preventive and therapeutic approaches are urgently needed (Kritsilis et al., 2018). Our research brings new perspectives to the treatment of aging and related diseases.

TGF-β and MMP-1 are secreted as one of the SASP factors, and they can induce senescent phenotype and age-related pathological conditions in an autocrine/paracrine manner. TGF-β promotes aging through inhibiting cell growth, induction of reactive oxygen species production, inhibition of telomerase activity, and impairment of DNA damage repair (Tominaga and Suzuki, 2019). Increased MMP-1 activity is an important indicator of cellular senescence and age-related skin changes (Lee et al., 2023). Our work showed that ABS decreased levels of TGF-β and MMP-1 to prevent age-related skin changes. However, the evaluation of skin-related effects in this study was predominantly based on measurements of epidermal thickness and histological observation of elastic fibers. Although these evaluations indicated improvements in aging-related skin phenotypes, incorporating more comprehensive metrics, such as collagen density and elastin quantification, would provide more robust evidence of skin-protective effects.

Among the several well-known hypotheses of aging, the most widely accepted theory is that aging is caused by oxidative stress (Salmon et al., 2010). The oxidative stress theory of aging elaborates that aerobic organisms experience chronic oxidative stress due to an imbalance between prooxidants and antioxidants under normal physiological conditions. This imbalance leads to the accumulation of oxidative damage to cellular macromolecules that increases during aging and contributes to a progressive decline in the function of cellular processes. Under this mechanistic framework, the regulation of oxidative stress may directly control the aging process (Yun et al., 2023). In this study, aging rats presented higher levels of MDA and lower activities of SOD and GSH-Px compared with control rats. Yet ABS receiving pulled down MDA content and enhanced activities of SOD and GSH-Px, suggesting that ABS reduced oxidative stress in aging rats. Notably, Vitamin B1, a crucial component of ABS, plays a significant role in the body’s carbohydrate and energy metabolism (Mikkelsen et al., 2024). By ameliorating aging-related metabolic disorders, it mitigates oxidative stress-induced damage and cellular fatigue, thereby synergistically enhancing the anti-aging effects of ABS. Despite Vitamin B1 being a marker for ABS quality control in the China Pharmacopoeia, its identification in this study was not feasible using UPLC-MS due to its water solubility and unique chemical properties.

Prior research has indicated that the expression of pro-inflammatory cytokines, such as IL-1β, IL-6, and TNF-α, is markedly elevated in aged tissues, and elevated levels of CRP are closely associated with the aging process and the onset of age-related diseases (Michaud et al., 2013). In contrast, the anti-inflammatory cytokine IL-10 plays a pivotal role in preserving organismal health by mitigating chronic inflammatory responses. Our findings revealed that ABS treatment significantly decreased the levels of pro-inflammatory molecules while concurrently elevating IL-10 levels in aging rats. This evidence strongly implies that ABS exerts its anti-aging effects through the modulation of inflammatory processes.

Preliminary evaluations of efficacy, encompassing behavioral tests, ELISA, and histopathology, indicated that the high-dose ABS group (ASH) demonstrated the most significant improvements in aging-related phenotypes. To elucidate the fundamental mechanisms underlying the optimal therapeutic effects of ABS and to minimize confounding influences from suboptimal doses, ASH group was concentrated in the omics analysis. Transcriptomic and metabolomic analysis results indicate that ABS exerts its anti-aging effects through the regulation of sphingolipid and arachidonic acid metabolism. In the present study, treatment with ABS in aging rats resulted in significant alterations in key sphingolipid metabolites and associated molecules: there was an increase in the levels of LPAR3, dihydro-S1P, Cer(d18:0/18:0), and Cer(d18:0/20:0), while the content of phytosphingosine was reduced. These modifications are consistent with the recognized roles of components within the sphingolipid pathway in neuroprotection and the regulation of aging, as supported by previous research. Lysophosphatidic acid (LPA) and its receptor are crucial for the development of the nervous system (Pitson and Pébay, 2009). The activation of LPAR3 enhances the expression of antioxidant enzymes, thereby reducing the accumulation of reactive oxygen species (ROS) and maintaining mitochondrial homeostasis (Chiang et al., 2022). In fibroblasts derived from patients with Hutchinson-Gilford progeria syndrome, downregulation of LPAR3 exacerbates ROS accumulation and cellular senescence, underscoring its potential role in mitigating senescence. (Chen et al., 2020). The identify of CYP2J10, CBR3, and LPAR3 as potential regulatory factors was based on integrated transcriptomic and metabolomic analyses. Their regulatory roles in ABS-mediated anti-aging effects require further verification through targeted functional experiments (e.g., gene overexpression, knockdown, or knockout studies).

Knockdown of S1PR1 increases apoptosis and decreases mitosis in neuroepithelial cells. (Mizugishi et al., 2005). Sphinganine 1-phosphate, a reduced form of sphingosine-1-phosphate (S1P), has been demonstrated to activate S1PR in neuronal progenitor cells (Callihan et al., 2012). Furthermore, dihydro-S1P has been shown to regulate the proliferation and differentiation of neuronal cells similarly to S1P. Notably, dihydro-S1P exhibits greater potency than S1P in activating Smad and inhibiting cAMP in progenitor cells (Callihan et al., 2012). The administration of ABS significantly increased plasma levels of dihydro-S1P and upregulated S1PR expression, although the latter was not statistically significant (Figure 10E). This suggests that ABS modulates S1PR signaling by regulating sphingolipid metabolism rather than altering S1PR expression.

Studies conducted both *in vivo* and *in vitro* have indicated that phytosphingosine effectively suppresses inflammatory responses and the expression of cytokines and chemokines by inhibiting the activation of NF-κB and NLRP3 (Montenegro-Burke et al., 2021; Zhao et al., 2023). Conversely, other studies have shown that phytosphingosine can promote inflammatory responses, oxidative stress, and induce apoptosis (Li et al., 2022; Takahashi et al., 2019). The physiological activities of phytosphingosine are thus varied, and its role in aged rats remains to be fully elucidated. ABS reduces phytosphingosine levels in aging rats, offering new insights into the physiological role of phytosphingosine.

An additional significant mechanism by which ABS exerts its effects is through the regulation of arachidonic acid metabolism. The administration of ABS resulted in notable alterations in the key DEGs and metabolites (CYP2J10, CBR3, 20-OH-LTB4, 20-OXO-LTE4) associated with arachidonic acid metabolism. The human gene CYP2J2, which is homologous to the rat gene CYP2J10, encodes a protein capable of oxidizing arachidonic acid to epoxyeicosatrienoic acids (EETs), thereby playing a potential role in the inflammatory process (Das et al., 2020; Wang et al., 2019). Inhibition of CYP2J2 is regarded as an effective strategy for suppressing inflammatory responses (Sisignano et al., 2020). Carbonyl reductase 3 (CBR3) catalyzes the conversion of prostaglandin E2 (PGE2) to PGF2α, both of which influence neuroinflammation through various signaling pathways (Glushakov et al., 2013; Woodling and Andreasson, 2016). The compound 20-OH-LTB4, a 20-hydroxy derivative of leukotriene B4, serves as a potent lipid chemoattractant that drives inflammatory responses (Di Gennaro and Haeggström, 2014), similar to leukotriene B4, which recruits leukocytes and induces inflammation (Dalli and Serhan, 2012). Conversely, 20-OXO-LTE4, a metabolite formed through the lipid oxidation of leukotriene E4, primarily modulates the cyclic adenosine monophosphate (cAMP) signaling pathway to exert anti-inflammatory effects (Steinke et al., 2014). Collectively, these findings, along with the observation that ABS downregulates CYP2J10, CBR3, 20-OH-LTB4 and increases the expression of 20-OXO-LTE4, suggest that ABS confers protection against aging in rats by modulating arachidonic acid metabolism and mitigating inflammatory responses.

This study employs an integrated approach combining metabolomics and transcriptomics to investigate the potential anti-aging mechanisms of ABS and to identify potential genes and associated metabolic changes, such as CYP2J10 and LPAR3. Although these genes have not yet been subjected to functional validation, their expression and related metabolites suggest they play a significant role in the anti-aging process and represent promising targets for further anti-aging research. While the study suggests ABS’s anti-aging efficacy and potential mechanisms, further validation using diverse aging models and bisexual animal models is necessary. The findings indicate that ABS alleviates aging-related neuronal loss, cognitive decline, and neuroinflammation, suggesting its potential in treating age-related neurodegenerative diseases.

## 5 Conclusion

In this study, ABS was found to mitigate skin aging and brain injury while ameliorating the abnormal activation of senescence-associated secretory phenotype (SASP) in rats exposed to D-galactose. The proposed anti-aging mechanism of ABS involves the inhibition of inflammation and oxidative stress by modulating arachidonic acid and sphingolipid metabolism through the regulation of CYP2J10/CBR3 and LPAR3 expression. This research provides novel insights into the prevention and treatment of aging and age-related diseases.

## Data Availability

The datasets presented in this study can be found in online repositories. The names of the repository/repositories and accession number(s) can be found in the article/Supplementary Material.
