# Supplementary material for: Anshen Bunao syrup as a potential anti-aging agent: mechanistic insights and pharmacological evidence
Source: Front Aging. 2026 Mar 10;7:1768671. doi: 10.3389/fragi.2026.1768671 (PMC13008913; doi:10.3389/fragi.2026.1768671)
Supplement: Supplementary file 1 [file Table1.docx]

Table S1 The statistical results for 281 differentially expressed genes

| **id** | **Symbol** | **CON-vs-MOD-log2fc** | **CON-vs-MOD -pvalue** | **MOD-vs-ASH-log2fc** | **MOD-vs-ASH -pvalue** |
| --- | --- | --- | --- | --- | --- |
| ENSRNOG00000000341 | Nid2 | -0.18372 | 0.571599 | 0.619463 | 0.011318 |
| ENSRNOG00000000384 | Atoh7 | -3.34864 | 0.139796 | 3.917538 | 0.043074 |
| ENSRNOG00000000521 | Cdkn1a | 0.695698 | 0.006856 | -0.53559 | 0.004197 |
| ENSRNOG00000001031 | Ocm | -1.55024 | 0.034969 | 1.418587 | 0.044137 |
| ENSRNOG00000001187 | Oasl | -2.09203 | 0.001652 | 0.586375 | 0.437254 |
| ENSRNOG00000001189 | Sik1 | -0.07714 | 0.986696 | 0.9445 | 0.00024 |
| ENSRNOG00000001242 | Gstt3 | 0.719376 | 7.77E-06 | -0.13041 | 0.730734 |
| ENSRNOG00000001251 | Grifin | 3.697373 | 0.004341 | -3.34994 | 7.32E-06 |
| ENSRNOG00000001284 | Uncx | 3.78595 | 0.00575 | -0.44188 | 0.48558 |
| ENSRNOG00000001300 | P2rx4 | -0.76797 | 1.99E-05 | 0.627252 | 0.004843 |
| ENSRNOG00000001392 | Lhx5 | 2.213691 | 0.009821 | -1.28816 | 0.094552 |
| ENSRNOG00000001701 | Cbr3 | 0.162286 | 0.467202 | -0.59817 | 0.029848 |
| ENSRNOG00000002217 | Plac8 | -1.35502 | 0.050614 | 1.486629 | 0.005359 |
| ENSRNOG00000002385 | Prg4 | -0.61165 | 0.076465 | 0.685157 | 0.023503 |
| ENSRNOG00000002592 | Rps6ka6 | 0.651903 | 0.03045 | -0.40607 | 0.139832 |
| ENSRNOG00000002607 | Sox9 | -0.07647 | 0.904457 | 0.811248 | 0.000869 |
| ENSRNOG00000002812 | Lhx1 | 2.696471 | 0.010053 | -1.42345 | 0.077658 |
| ENSRNOG00000003105 | Kif19 | -0.79109 | 0.023882 | 0.358871 | 0.184818 |
| ENSRNOG00000003201 | Rps4x | -0.60853 | 0.001874 | 0.261439 | 0.127504 |
| ENSRNOG00000003300 | Btg2 | -0.56864 | 0.32329 | 1.267226 | 0.009512 |
| ENSRNOG00000003553 | Efemp1 | -0.50463 | 0.056191 | 0.676386 | 0.005194 |
| ENSRNOG00000003569 | Gabra6 | 2.943993 | 0.004886 | -1.248 | 0.166811 |
| ENSRNOG00000003666 | Jchain | 0.986197 | 4.20E-05 | -1.20593 | 4.09E-08 |
| ENSRNOG00000003745 | Atf3 | -1.06909 | 0.143611 | 2.991595 | 0.002578 |
| ENSRNOG00000003785 | Usp43 | -0.30542 | 0.344765 | 0.648375 | 0.01916 |
| ENSRNOG00000003807 | Wnt9b | 0.787592 | 0.004377 | -0.85335 | 0.011707 |
| ENSRNOG00000003845 | Wnt3 | 0.820982 | 0.03549 | -0.79884 | 0.066094 |
| ENSRNOG00000003927 | Cd55 | 0.956471 | 0.003562 | -0.69869 | 0.054313 |
| ENSRNOG00000004009 | Xpnpep2 | -1.64454 | 0.114876 | 1.881193 | 0.049982 |
| ENSRNOG00000004311 | Gpr182 | -0.35776 | 0.13864 | 0.723533 | 0.002499 |
| ENSRNOG00000004317 | Vipr2 | 0.490484 | 0.037359 | -0.65003 | 0.014433 |
| ENSRNOG00000004378 | Abca5 | -0.25415 | 0.556148 | 0.890191 | 0.01275 |
| ENSRNOG00000004642 | Lmx1a | 1.618753 | 0.02723 | -1.19617 | 0.045272 |
| ENSRNOG00000004679 | Fign | 0.666576 | 0.025234 | -0.11978 | 0.638359 |
| ENSRNOG00000004713 | Kcnj16 | 0.598781 | 0.000482 | -0.37961 | 0.044575 |
| ENSRNOG00000004754 | Tc2n | 0.598815 | 0.188474 | -1.06392 | 0.048791 |
| ENSRNOG00000005580 | Itgb4 | -0.39179 | 0.090989 | 0.737004 | 0.000103 |
| ENSRNOG00000005659 | Aurkb | -0.51534 | 0.619851 | 2.087689 | 0.002442 |
| ENSRNOG00000005679 | Fap | -0.86386 | 0.026598 | 0.383636 | 0.34546 |
| ENSRNOG00000005947 | Pde6h | -7.38658 | 0.444714 | 9.084808 | 0.03613 |
| ENSRNOG00000005960 | RGD1311744 | 0.601372 | 0.000309 | -0.14671 | 0.297703 |
| ENSRNOG00000006324 | - | -0.48705 | 0.111122 | 0.851016 | 0.001996 |
| ENSRNOG00000006333 | AABR07065789.1 | 1.760807 | 5.53E-05 | -2.86048 | 0.012558 |
| ENSRNOG00000007084 | Marchf10 | -0.68639 | 0.292316 | 1.048919 | 0.016729 |
| ENSRNOG00000007338 | Fbln2 | -0.41161 | 0.063061 | 0.619139 | 0.002102 |
| ENSRNOG00000007552 | Arhgap36 | 1.182247 | 0.000257 | -0.17895 | 0.421614 |
| ENSRNOG00000007607 | Nr4a1 | -0.10556 | 0.920343 | 0.997095 | 0.012612 |
| ENSRNOG00000007608 | Fezf1 | 2.549276 | 0.035483 | -0.18566 | 0.793093 |
| ENSRNOG00000007675 | Gpr63 | 0.595652 | 0.000227 | -0.49162 | 0.005428 |
| ENSRNOG00000007830 | Apold1 | -0.54324 | 0.650251 | 2.011975 | 0.013916 |
| ENSRNOG00000008602 | Steap4 | 1.767177 | 9.73E-05 | -0.6334 | 0.190485 |
| ENSRNOG00000008855 | Gjb2 | -0.34092 | 0.115967 | 0.629223 | 0.001329 |
| ENSRNOG00000008936 | Map3k6 | 0.254503 | 0.461872 | -0.98122 | 0.01533 |
| ENSRNOG00000009431 | Tbc1d4 | 0.325854 | 0.021771 | -0.67068 | 3.31E-05 |
| ENSRNOG00000009434 | RGD1310507 | -0.8759 | 0.023345 | 1.187324 | 0.001119 |
| ENSRNOG00000009448 | Papln | 0.787244 | 0.025277 | -0.62864 | 0.034075 |
| ENSRNOG00000009450 | Hcn4 | 0.642421 | 0.00177 | -0.49534 | 0.014071 |
| ENSRNOG00000009565 | Pdk4 | 1.065213 | 0.023434 | -0.57965 | 8.51E-05 |
| ENSRNOG00000010079 | Ca3 | -2.1181 | 0.040303 | 2.093216 | 1 |
| ENSRNOG00000010121 | Lef1 | 0.644946 | 0.002664 | -0.31171 | 0.178338 |
| ENSRNOG00000010128 | Slc27a2 | 0.947976 | 0.04971 | -1.19583 | 0.010734 |
| ENSRNOG00000010165 | Tnfaip2 | -0.49274 | 0.13385 | 0.696795 | 0.021547 |
| ENSRNOG00000010357 | Lhx9 | 1.588005 | 0.000643 | -1.12951 | 0.01367 |
| ENSRNOG00000010423 | Misp | -1.15044 | 0.249533 | 1.666654 | 0.042567 |
| ENSRNOG00000010666 | Ccn5 | -0.17971 | 0.679186 | 0.885775 | 0.007885 |
| ENSRNOG00000010797 | Esm1 | -4.29502 | 0.001738 | 0.65966 | 0.774729 |
| ENSRNOG00000010977 | Igfbp6 | -0.46716 | 0.12308 | 0.758895 | 0.002838 |
| ENSRNOG00000011533 | Irx3 | 2.412353 | 0.017571 | -1.55013 | 0.088317 |
| ENSRNOG00000011750 | Fam180a | -0.72648 | 0.018613 | 1.106933 | 8.51E-05 |
| ENSRNOG00000011936 | Abhd14a | -0.50663 | 0.049996 | 0.638726 | 0.00462 |
| ENSRNOG00000012235 | Ppp1r17 | 1.539213 | 0.006564 | -1.54925 | 0.010029 |
| ENSRNOG00000012283 | Chrna6 | 3.609888 | 0.0618 | -2.92429 | 0.004233 |
| ENSRNOG00000012448 | Chrnb3 | 0.903852 | 0.046597 | -0.8403 | 0.066625 |
| ENSRNOG00000012512 | Nexn | 0.34904 | 0.094042 | -0.58872 | 0.012773 |
| ENSRNOG00000012575 | Fat2 | 1.261125 | 0.020891 | -0.88085 | 0.125901 |
| ENSRNOG00000012742 | Irx2 | 2.789026 | 0.000746 | -1.6424 | 0.030425 |
| ENSRNOG00000012817 | Lrrc17 | -0.70354 | 0.023653 | 0.628881 | 0.0276 |
| ENSRNOG00000012980 | Hyls1 | -0.65478 | 0.046051 | 0.643935 | 0.047559 |
| ENSRNOG00000013092 | Lonrf3 | 0.653612 | 0.00187 | -0.09975 | 0.548999 |
| ENSRNOG00000013209 | Barhl1 | 3.441178 | 0.016208 | -2.50391 | 0.030155 |
| ENSRNOG00000013330 | Cdhr1 | -0.67166 | 0.001485 | 0.142247 | 0.395246 |
| ENSRNOG00000013399 | Minar1 | 0.710368 | 0.014762 | -0.31297 | 0.237835 |
| ENSRNOG00000013426 | Mrgprf | -0.37086 | 0.346268 | 0.892184 | 0.004945 |
| ENSRNOG00000013588 | Glra1 | 1.170669 | 0.047564 | -0.86964 | 0.189295 |
| ENSRNOG00000013768 | Defb1 | -1.13519 | 0.177821 | 1.601491 | 0.022795 |
| ENSRNOG00000013829 | Chrna3 | 0.840255 | 0.000797 | -1.09539 | 0.000194 |
| ENSRNOG00000013887 | Adra2b | 1.076069 | 0.004868 | -0.73072 | 0.08918 |
| ENSRNOG00000013928 | Dsp | 0.65709 | 0.024591 | -0.66526 | 0.035351 |
| ENSRNOG00000014011 | Dll4 | -0.74184 | 0.02499 | 0.99742 | 0.002353 |
| ENSRNOG00000014372 | Gjb3 | -1.68442 | 0.024043 | 0.987422 | 0.194366 |
| ENSRNOG00000014385 | Wnt2b | 1.138297 | 0.04118 | -0.37752 | 0.596326 |
| ENSRNOG00000014452 | Zfhx3 | 0.634019 | 0.001236 | -0.29934 | 0.178589 |
| ENSRNOG00000014644 | Zic1 | 0.717432 | 1.58E-05 | -0.16238 | 0.33143 |
| ENSRNOG00000014751 | Ret | 1.265646 | 5.65E-08 | -0.94554 | 0.000218 |
| ENSRNOG00000014867 | Synpo2 | 1.171313 | 0.031271 | -0.95177 | 0.142768 |
| ENSRNOG00000014871 | Zic4 | 1.013456 | 0.034064 | -0.43333 | 0.618494 |
| ENSRNOG00000015071 | Zim1 | 0.913486 | 0.019985 | -0.30785 | 0.335725 |
| ENSRNOG00000015078 | Ifitm3 | -0.62216 | 0.028477 | 0.421497 | 0.125014 |
| ENSRNOG00000015160 | Gem | -0.41038 | 0.228735 | 0.838214 | 0.00195 |
| ENSRNOG00000015402 | Guca1a | -4.21471 | 0.025753 | 2.477047 | 0.304658 |
| ENSRNOG00000016011 | Plekhg1 | 0.851536 | 2.40E-05 | -0.79718 | 0.00053 |
| ENSRNOG00000016021 | Lims2 | 0.49521 | 0.029664 | -0.78781 | 0.001762 |
| ENSRNOG00000016102 | Ebf3 | 1.562624 | 0.005166 | -0.93459 | 0.078748 |
| ENSRNOG00000016147 | Slc17a6 | 1.36646 | 0.002395 | -1.04397 | 0.025976 |
| ENSRNOG00000016164 | Fcrl2 | 2.69454 | 0.039297 | -0.01187 | 0.992875 |
| ENSRNOG00000016243 | Casq2 | 0.836546 | 0.031781 | -0.59956 | 0.192054 |
| ENSRNOG00000016346 | Prkcd | 1.055215 | 0.024832 | -0.66304 | 0.258283 |
| ENSRNOG00000016525 | Susd3 | -0.80473 | 0.017411 | 0.486428 | 0.136481 |
| ENSRNOG00000016622 | Ankra2 | 1.176477 | 0.000163 | -0.63437 | 0.028798 |
| ENSRNOG00000016917 | Clcn1 | 1.63653 | 0.013968 | -1.89579 | 0.007865 |
| ENSRNOG00000016977 | Calb2 | 0.729268 | 1.17E-05 | -0.45748 | 0.222276 |
| ENSRNOG00000017198 | Hif3a | 2.446145 | 0.000188 | -1.64748 | 0.00272 |
| ENSRNOG00000017302 | Slc6a3 | 6.133364 | 0.007052 | -6.12716 | 0.000928 |
| ENSRNOG00000017409 | Wnt6 | -0.14591 | 0.794432 | 1.097116 | 0.001822 |
| ENSRNOG00000017872 | - | -1.40902 | 8.74E-11 | 0.637064 | 0.00396 |
| ENSRNOG00000017893 | Baiap3 | 0.665974 | 0.00147 | -0.12803 | 0.420977 |
| ENSRNOG00000018191 | Oprm1 | 0.98804 | 0.01179 | -0.50486 | 0.174992 |
| ENSRNOG00000018215 | Slc22a6 | -0.52663 | 0.021474 | 0.791286 | 0.000601 |
| ENSRNOG00000018371 | Tubb6 | -0.54211 | 0.141721 | 0.632509 | 0.026891 |
| ENSRNOG00000018379 | Zfp688 | -0.71004 | 0.005754 | 0.413346 | 0.09231 |
| ENSRNOG00000018969 | Gpatch4 | 0.735681 | 5.94E-05 | -0.1696 | 0.33142 |
| ENSRNOG00000019141 | Ch25h | -0.18958 | 0.690177 | 0.840898 | 0.00394 |
| ENSRNOG00000019202 | Pvr | -0.5594 | 0.199369 | 0.721088 | 0.048718 |
| ENSRNOG00000019283 | P2ry2 | -0.15565 | 0.786849 | 0.669199 | 0.020079 |
| ENSRNOG00000019336 | Gata3 | 4.393522 | 0.001579 | -5.18202 | 0.00136 |
| ENSRNOG00000019342 | Sult1a1 | 0.680638 | 0.000174 | -0.15911 | 0.391304 |
| ENSRNOG00000019495 | Gbx2 | 1.263612 | 0.015943 | -0.28293 | 0.608799 |
| ENSRNOG00000019584 | Dlk1 | 0.9088 | 0.001436 | -0.4329 | 0.080408 |
| ENSRNOG00000019613 | Syt9 | 0.599688 | 0.000361 | -0.55832 | 0.029564 |
| ENSRNOG00000019648 | Col6a3 | -0.26165 | 0.375188 | 0.592037 | 0.026354 |
| ENSRNOG00000019718 | Galnt15 | 0.677064 | 0.011411 | -0.83998 | 0.000997 |
| ENSRNOG00000020009 | Npas4 | -0.6631 | 0.157996 | 0.977064 | 5.18E-05 |
| ENSRNOG00000020054 | Pcdhb15 | 0.081832 | 0.631174 | -0.59468 | 0.022588 |
| ENSRNOG00000020151 | Cdh1 | -0.6964 | 0.016531 | 0.796774 | 0.00539 |
| ENSRNOG00000020410 | Th | 2.634986 | 0.001109 | -2.00719 | 0.009792 |
| ENSRNOG00000020485 | Vav3 | 0.512736 | 0.022309 | -0.74902 | 0.000718 |
| ENSRNOG00000020503 | Cbln3 | 5.021784 | 0.00256 | -3.29243 | 0.024951 |
| ENSRNOG00000020598 | Kcnk6 | -0.57543 | 0.154486 | 0.874679 | 0.010919 |
| ENSRNOG00000020938 | Ppp1r15a | -0.33664 | 0.223268 | 0.637892 | 0.011694 |
| ENSRNOG00000021521 | Chst5 | 0.681602 | 0.045639 | -0.50057 | 0.101991 |
| ENSRNOG00000021814 | Tnfrsf25 | -1.89776 | 0.042314 | 1.33232 | 0.154452 |
| ENSRNOG00000022337 | Slitrk6 | 1.674821 | 1.48E-08 | -1.27148 | 2.21E-05 |
| ENSRNOG00000022697 | Clec14a | -0.36417 | 0.29743 | 0.709237 | 0.018909 |
| ENSRNOG00000022839 | Ifit3 | -1.00684 | 0.002427 | 1.293229 | 0.001577 |
| ENSRNOG00000023561 | Ano2 | -0.67592 | 0.027186 | 0.418965 | 0.140152 |
| ENSRNOG00000023863 | Gpr139 | 0.784635 | 0.252204 | -1.83303 | 0.026407 |
| ENSRNOG00000023870 | Sh2d7 | 3.074347 | 0.026661 | -1.16008 | 0.412739 |
| ENSRNOG00000024277 | Card11 | -0.48768 | 0.12521 | 0.648782 | 0.021402 |
| ENSRNOG00000024506 | - | 0.713623 | 0.025359 | -0.59822 | 0.055561 |
| ENSRNOG00000024707 | Tp73 | 0.628398 | 0.026537 | -0.36505 | 0.30184 |
| ENSRNOG00000025121 | Pla2g3 | 1.224587 | 0.001887 | -0.29291 | 0.434142 |
| ENSRNOG00000025670 | Shisa3 | -0.88332 | 0.002201 | 0.626728 | 0.027943 |
| ENSRNOG00000025848 | Sspo | 2.553677 | 0.001818 | -3.28115 | 0.000185 |
| ENSRNOG00000026400 | AABR07020999.1 | 10.21513 | 0.002448 | -1.13298 | 0.353336 |
| ENSRNOG00000026914 | Dnah1 | -0.07525 | 0.968777 | 0.879829 | 0.016037 |
| ENSRNOG00000027103 | Gpr151 | 1.890789 | 0.02467 | -1.41576 | 0.040735 |
| ENSRNOG00000028064 | Fhad1 | 1.652504 | 0.014681 | -1.02046 | 0.107389 |
| ENSRNOG00000028311 | Cnpy1 | 3.208919 | 0.041115 | -1.43296 | 0.215246 |
| ENSRNOG00000028456 | Ush1g | -1.16082 | 0.007348 | 0.677051 | 0.133769 |
| ENSRNOG00000028622 | Pnpla1 | -2.35035 | 0.001124 | 1.084921 | 0.101651 |
| ENSRNOG00000029128 | Cyp2d5 | -8.20131 | 0.014736 | 7.779172 | 0.037813 |
| ENSRNOG00000029242 | Plekho2 | -0.29374 | 0.069372 | 0.661606 | 2.89E-06 |
| ENSRNOG00000029342 | Scn7a | 1.471998 | 0.002498 | -1.21131 | 0.010928 |
| ENSRNOG00000029841 | Cdh19 | 0.667259 | 0.033752 | -0.33304 | 0.255154 |
| ENSRNOG00000029980 | Zbtb16 | 0.230003 | 0.101415 | -0.59523 | 0.000489 |
| ENSRNOG00000030332 | Ighg1 | 0.635609 | 0.275666 | -1.62775 | 0.004067 |
| ENSRNOG00000030481 | - | 0.615896 | 0.014088 | -0.59398 | 0.035128 |
| ENSRNOG00000030812 | AABR07065781.1 | 0.656323 | 0.246405 | -2.78029 | 1.96E-05 |
| ENSRNOG00000030927 | Grid2ip | 1.097346 | 0.000231 | -0.53005 | 0.120893 |
| ENSRNOG00000031483 | AABR07071482.1 | -0.84369 | 0.178159 | 1.41467 | 0.024087 |
| ENSRNOG00000032596 | RT1-T24-1 | -0.84647 | 0.005878 | 0.471731 | 0.177711 |
| ENSRNOG00000033192 | Osmr | 0.294621 | 0.294918 | -0.81824 | 0.01086 |
| ENSRNOG00000033220 | Oas1k | -0.61518 | 0.031628 | 0.026468 | 0.934157 |
| ENSRNOG00000033734 | Tnnt2 | -0.22497 | 0.455667 | 0.697543 | 0.00232 |
| ENSRNOG00000033748 | AABR07060610.1 | 0.748423 | 0.012142 | -0.39946 | 0.480091 |
| ENSRNOG00000033906 | Zfp667 | 0.547979 | 0.103352 | -0.72916 | 0.027996 |
| ENSRNOG00000034161 | Cox6b1-ps1 | 4.315202 | 0.032957 | -0.27794 | 0.713925 |
| ENSRNOG00000037148 | Adap2 | 0.408948 | 0.224075 | -0.79194 | 0.028447 |
| ENSRNOG00000037190 | Cd101 | 0.801286 | 0.009441 | -0.36257 | 0.194002 |
| ENSRNOG00000037600 | Sim1 | 2.516483 | 0.002055 | -0.67274 | 0.193548 |
| ENSRNOG00000037801 | - | -2.74478 | 0.029504 | 1.799087 | 0.14246 |
| ENSRNOG00000037919 | - | 0.657116 | 0.002099 | -0.15985 | 0.268272 |
| ENSRNOG00000038074 | - | -0.37356 | 0.70221 | 1.489081 | 0.024514 |
| ENSRNOG00000038999 | RT1-CE11 | -0.86338 | 0.026442 | 0.069609 | 0.732545 |
| ENSRNOG00000039152 | Frem3 | 0.753389 | 0.094508 | -0.85219 | 0.016393 |
| ENSRNOG00000039832 | Gpr12 | -0.61483 | 0.005258 | 0.566623 | 0.008013 |
| ENSRNOG00000042224 | Cyp2j10 | 0.315738 | 0.132779 | -0.63712 | 0.00743 |
| ENSRNOG00000043451 | Spp1 | 1.46272 | 0.029819 | -1.20489 | 0.078437 |
| ENSRNOG00000043465 | Arc | -0.06862 | 0.996766 | 0.830278 | 0.036605 |
| ENSRNOG00000045743 | Etnppl | 0.870729 | 0.001439 | -0.49222 | 0.046682 |
| ENSRNOG00000045831 | Lmod2 | 2.802061 | 0.032134 | -1.09028 | 0.312097 |
| ENSRNOG00000046326 | Rp1l1 | -3.06054 | 0.005203 | 0.280108 | 0.804918 |
| ENSRNOG00000046428 | Lrrc75b | 0.740083 | 0.034897 | -0.455 | 0.139186 |
| ENSRNOG00000047276 | - | -0.39848 | 0.492309 | 1.232374 | 0.00956 |
| ENSRNOG00000047446 | Foxc2 | -0.15595 | 0.774249 | 0.90506 | 0.003008 |
| ENSRNOG00000047884 | AABR07001923.1 | 0.641163 | 0.635364 | -4.39232 | 0.007734 |
| ENSRNOG00000048230 | - | 0.77776 | 0.249474 | -1.60215 | 0.026708 |
| ENSRNOG00000048356 | RGD1308750 | -6.66378 | 0.0244 | 6.535181 | 0.001437 |
| ENSRNOG00000048924 | Islr | -0.46422 | 0.036116 | 0.635484 | 0.006884 |
| ENSRNOG00000049232 | Tcf7l2 | 1.523746 | 0.009924 | -1.09969 | 0.088693 |
| ENSRNOG00000049829 | AABR07060872.1 | 0.993747 | 1.34E-12 | -1.21411 | 6.26E-10 |
| ENSRNOG00000049994 | Ifi44l | -1.25343 | 0.031173 | 0.472902 | 0.475747 |
| ENSRNOG00000050006 | Agtr2 | 2.129681 | 0.02908 | -0.86742 | 0.221483 |
| ENSRNOG00000050091 | Efcab1 | 1.405505 | 0.002227 | -0.40637 | 0.329885 |
| ENSRNOG00000050465 | AABR07041109.1 | 1.41502 | 0.008729 | -0.53976 | 0.202305 |
| ENSRNOG00000050792 | Tnfaip6 | 0.26633 | 0.381032 | -0.70195 | 0.040659 |
| ENSRNOG00000051739 | AABR07061001.1 | 3.330711 | 0.003179 | -5.6087 | 0.000202 |
| ENSRNOG00000053384 | Bmp7 | -0.41114 | 0.062162 | 0.689398 | 0.002092 |
| ENSRNOG00000053527 | AABR07034736.1 | 10.30492 | 0.039767 | -10.3049 | 0.031093 |
| ENSRNOG00000053753 | AABR07070307.1 | -0.64495 | 0.004323 | 0.660953 | 0.0041 |
| ENSRNOG00000054063 | Naa80 | -0.72059 | 0.01354 | 0.44255 | 0.137749 |
| ENSRNOG00000054375 | Nhlh2 | 2.16692 | 0.002277 | -0.96552 | 0.109002 |
| ENSRNOG00000055078 | Cyp4b1 | 1.423704 | 0.003365 | -1.21839 | 0.032721 |
| ENSRNOG00000056052 | AABR07060980.1 | 0.457033 | 0.687098 | -9.55331 | 0.014876 |
| ENSRNOG00000056457 | Gpd1 | 1.102841 | 5.54E-17 | -0.20152 | 0.166261 |
| ENSRNOG00000056493 | Mybpc1 | 0.615565 | 0.037194 | -0.80872 | 0.022516 |
| ENSRNOG00000056580 | En1 | 3.245237 | 0.01158 | -2.87559 | 0.013241 |
| ENSRNOG00000057162 | RGD1564409 | 0.224409 | 0.4696 | -0.99321 | 0.0467 |
| ENSRNOG00000057256 | Slc19a3 | 0.818372 | 0.000646 | -0.61163 | 0.00766 |
| ENSRNOG00000057557 | Prlr | 0.894164 | 0.441174 | -1.2932 | 0.018017 |
| ENSRNOG00000059447 | AC109901.2 | 3.459862 | 0.027864 | -2.46223 | 0.049046 |
| ENSRNOG00000059702 | Tex9 | 0.687208 | 0.028646 | -0.57416 | 0.08601 |
| ENSRNOG00000059793 | Rab37 | 1.13031 | 0.001683 | -0.60042 | 0.330279 |
| ENSRNOG00000059947 | Sdc1 | 0.911655 | 0.019874 | -0.57349 | 0.133636 |
| ENSRNOG00000061320 | - | 1.166225 | 0.479014 | -1.28328 | 0.000231 |
| ENSRNOG00000061376 | Psca | 2.541254 | 0.047643 | -1.47458 | 0.117417 |
| ENSRNOG00000062245 | Ndufa10l1 | -0.67379 | 0.339207 | 1.125461 | 0.03111 |
| ENSRNOG00000062357 | - | -10.0616 | 0.043459 | 0 | 1 |
| ENSRNOG00000063087 | - | -0.27411 | 0.883224 | 1.343846 | 0.028051 |
| ENSRNOG00000063112 | - | -3.77259 | 1 | 8.639039 | 0.029128 |
| ENSRNOG00000063422 | - | 10.59712 | 0.004363 | -10.5971 | 0.003352 |
| ENSRNOG00000063523 | - | 11.41978 | 0.000254 | -11.4198 | 0.000191 |
| ENSRNOG00000063713 | - | 1.295308 | 0.166902 | -11.0136 | 0.000344 |
| ENSRNOG00000063806 | - | -4.09055 | 0.042675 | 3.615245 | 0.073331 |
| ENSRNOG00000063889 | - | 2.331976 | 0.029611 | -0.54583 | 0.509091 |
| ENSRNOG00000064003 | - | 1.512037 | 0.154787 | -3.49796 | 0.008773 |
| ENSRNOG00000064041 | - | 0.180669 | 0.756578 | -3.24689 | 0.00011 |
| ENSRNOG00000064048 | - | -0.52132 | 0.651406 | 2.07044 | 0.035142 |
| ENSRNOG00000064061 | - | 11.20335 | 0.000132 | -2.29846 | 0.032604 |
| ENSRNOG00000064204 | Lpar3 | -0.04461 | 0.99094 | 1.025613 | 0.031862 |
| ENSRNOG00000064589 | - | 1.071992 | 0.013759 | -1.58728 | 0.000102 |
| ENSRNOG00000064825 | - | 2.186878 | 0.005011 | -1.14477 | 0.113106 |
| ENSRNOG00000064836 | - | 3.752131 | 0.025069 | -5.35204 | 7.58E-05 |
| ENSRNOG00000064873 | - | 2.288976 | 0.005147 | -3.0879 | 0.000533 |
| ENSRNOG00000064886 | - | 0.695595 | 0.480066 | -3.30964 | 0.021613 |
| ENSRNOG00000064887 | - | 0.235895 | 0.374049 | -0.58789 | 0.040476 |
| ENSRNOG00000065181 | - | -3.36891 | 0.007899 | 3.759436 | 0.000638 |
| ENSRNOG00000065459 | - | -10.5353 | 0.042068 | 9.902375 | 0.072634 |
| ENSRNOG00000065866 | - | 2.964621 | 0.046639 | -0.76883 | 0.461394 |
| ENSRNOG00000065952 | - | 0.133027 | 0.848311 | -3.13562 | 0.035842 |
| ENSRNOG00000066203 | - | 4.178996 | 0.020765 | -2.5364 | 0.075224 |
| ENSRNOG00000066227 | - | 0.987028 | 0.005092 | -0.17724 | 0.494121 |
| ENSRNOG00000066431 | - | 3.638236 | 0.003895 | -1.87123 | 0.057306 |
| ENSRNOG00000066785 | - | 1.065181 | 0.018313 | -1.07944 | 0.047941 |
| ENSRNOG00000066999 | Gm48552 | -1.54561 | 0.001339 | 0.002229 | 0.944637 |
| ENSRNOG00000067025 | - | 1.252047 | 0.278853 | -3.83787 | 0.014851 |
| ENSRNOG00000067072 | - | -0.87612 | 0.015749 | 0.799972 | 0.05449 |
| ENSRNOG00000067139 | Rpl10l | -1.01634 | 0.004021 | 0.485925 | 0.241839 |
| ENSRNOG00000067278 | - | 3.408712 | 0.048362 | -1.10128 | 0.359692 |
| ENSRNOG00000068056 | - | 2.339888 | 0.053048 | -10.6345 | 0.000677 |
| ENSRNOG00000068063 | Rnaseh2a | 0.544231 | 0.367805 | -1.49333 | 0.024704 |
| ENSRNOG00000068331 | RGD1564941 | -1.20772 | 0.012104 | 0.283366 | 0.605086 |
| ENSRNOG00000068351 | - | -0.81539 | 0.033297 | 0.469027 | 0.178003 |
| ENSRNOG00000068563 | - | 0.879478 | 0.026465 | -0.40222 | 0.249458 |
| ENSRNOG00000068610 | - | 3.710461 | 0.030952 | -1.36446 | 0.284258 |
| ENSRNOG00000068619 | - | 4.347351 | 0.001228 | -0.20258 | 0.702245 |
| ENSRNOG00000068760 | - | -1.27801 | 0.199818 | 1.756365 | 0.036819 |
| ENSRNOG00000069024 | - | -0.17238 | 0.859949 | 0.639341 | 0.00345 |
| ENSRNOG00000069088 | - | 0.301907 | 0.797597 | -10.0023 | 0.005349 |
| ENSRNOG00000069319 | - | -1.77036 | 0.259791 | 2.77437 | 0.026372 |
| ENSRNOG00000069375 | Kctd12b | 0.810131 | 0.026107 | -0.17891 | 0.502433 |
| ENSRNOG00000069405 | - | 1.951969 | 0.027615 | -5.16142 | 0.000528 |
| ENSRNOG00000069536 | - | 3.827511 | 0.036236 | -1.11702 | 0.352281 |
| ENSRNOG00000070091 | - | 1.459698 | 0.075406 | -3.93637 | 0.000968 |
| ENSRNOG00000070169 | - | 10.42626 | 0.005348 | -4.22987 | 0.016748 |
| ENSRNOG00000070540 | - | 0.769774 | 0.254187 | -2.01232 | 0.024393 |
| ENSRNOG00000070810 | - | 2.933552 | 0.002696 | -2.23534 | 0.020168 |
| ENSRNOG00000070982 | - | -0.49441 | 0.577085 | 1.828324 | 0.014552 |
| ENSRNOG00000071034 | - | 0.266089 | 0.368839 | -0.97008 | 0.005518 |
| ENSRNOG00000071163 | - | 0.191158 | 0.768196 | -2.64053 | 0.034961 |
| ENSRNOG00000071189 | - | 1.05625 | 0.297227 | -3.60926 | 0.020107 |

Notes: CON, healthy control group; MOD, aging model group; ASH, *Anshen Bunao Syrup* high-dose administratio group; FC, fold change
